# Alpha-neurexins in health and disease

**DOI:** 10.3389/fnmol.2025.1716782

**Published:** 2025-11-17

**Authors:** Nicolas Chofflet, Manni Wang, Mathilde Chofflet, Hideto Takahashi

**Affiliations:** 1 Synapse Development and Plasticity Research Unit, Institut de Recherches Cliniques de Montréal, Montréal, QC, Canada; 2 Integrated Program in Neuroscience, McGill University, Montreal, QC, Canada; 3 UFR Santé, Université de Caen Normandie, Caen, France; 4 Department of Medicine, Université de Montréal, Montréal, QC, Canada; 5 Division of Experimental Medicine, McGill University, Montréal, QC, Canada

**Keywords:** alpha-neurexins, synapse, neuropsychiatric disorders, IgSF21, neurexophilin, hevin, CaVα2δ, dystroglycan

## Abstract

Alpha-neurexins (α-Nrxns) are synaptic adhesion molecules that play crucial roles in synapse organization, specificity, and function. This review provides a comprehensive overview of α-Nrxns, covering their gene organization, molecular architecture, and roles in both physiological and pathological contexts. We begin by detailing the unique structural properties of α-Nrxns, particularly their large extracellular regions and complex alternative splicing, which facilitate diverse trans-synaptic interactions. We then examine their critical roles in regulating presynaptic neurotransmitter release, postsynaptic receptor function, and overall synaptic organization. While deletion of α-Nrxns in mice results in only modest morphological brain abnormalities, it causes profound deficits in synaptic function, underscoring their role in fine-tuning neural circuit activity in a context-dependent manner. We also explore how specific α-Nrxn ligands such as neurexophilins or IgSF21 contribute to synaptic diversity. Furthermore, we discuss emerging evidence linking α-NRXNs to various neurodevelopmental and psychiatric disorders, including autism spectrum disorder, schizophrenia, and intellectual disability. These links are supported by both genetic association studies and behavioral analyses in α-Nrxn mutant mice, which exhibit phenotypes that partially mirror symptoms observed in human disorders. Finally, we highlight recent advances in human induced pluripotent stem cell (hiPSC)-derived neuronal models, which offer powerful platforms to investigate α-NRXN-associated disease mechanisms at the cellular level. These models enable the study of patient-specific neurobiological alterations and support the development of targeted therapeutic strategies. Collectively, this review emphasizes the pivotal role of α-Nrxns in maintaining synaptic integrity and demonstrates how their dysfunction contributes to a broad spectrum of brain disorders, providing valuable insights for future translational research.

## 1 Introduction

Neurexins (Nrxns) are pleiotropic cell adhesion molecules essential for synaptic organization and function (Sudhof, 2017; Gomez et al., 2021). In mammals, three *Nrxn* genes generate long α- and short β-isoforms through alternative promoter usage (Reissner et al., 2013). In humans, genetic alterations in *NRXN* genes, particularly within α-coding regions, have been repeatedly linked to psychiatric disorders (Bena et al., 2013; Kasem et al., 2018; Hu et al., 2019; Castronovo et al., 2020; Cooper et al., 2024). Despite over three decades of intensive research, the specific and overlapping roles of α- and β-Nrxns in synaptic connectivity remain poorly understood. This gap in knowledge is due to the lack of side-by-side, systematic analyses evaluating the contribution of each isoform to defined biological processes. This review summarizes current knowledge on α-Nrxns, emphasizing their unique structural features, functional roles, and ligand repertoire in comparison to β-Nrxns in both health and disease.

## 2 Gene organization and molecular architecture of Nrxns

In vertebrates, Nrxns are encoded by three genes (*Nrxn1, Nrxn2*, and *Nrxn3*), each regulated by two independent promoters that drive the production of long α-Nrxns and shorter β-Nrxns (Ushkaryov et al., 1992, 1994; Ushkaryov and Sudhof, 1993; Gomez et al., 2021). Additionally, the *Nrxn1* gene contains a third promoter responsible for expressing the shortest isoform, Nrxn1γ (Yan et al., 2015; Sterky et al., 2017). In humans, the *NRXN* genes rank among the largest in the genome: *NRXN1* spans approximately 1,108.4 kb with 24 exons, *NRXN2* spans 106.4 kb with 23 exons, and *NRXN3* spans 1,618.5 kb with 24 exons (Tabuchi and Sudhof, 2002). These genes are located at distinct chromosomal loci: *NRXN1* at 2p16.3, *NRXN2* at 11q13.1, and *NRXN3* at 14q24–q31.1. Phylogenetic analysis of *Nrxn* genes indicates that *Nrxn1* and *Nrxn3* are more closely related to each other than to *Nrxn2*, suggesting that *Nrxn2* diverged earlier from a common ancestor of *Nrxn1* and *Nrxn3* (Treutlein et al., 2014). In invertebrates such as *Drosophila melanogaster* and *Caenorhabditis elegans*, a single shorter *Nrxn* gene, homologous to mammalian *Nrxn1*, produces both the long α and short γ isoforms (Tabuchi and Sudhof, 2002; Haklai-Topper et al., 2011). Notably, the presence of a single α-Nrxn isoform in early diverging metazoans, including Ctenophora, Porifera and Placozoa, suggests that α-Nrxn represents the ancestral form of the Nrxn family (Guzman et al., 2024).

Structurally, the Nrnxs are all type I single transmembrane proteins with distinct extracellular regions followed by a conserved juxtamembranous stalk, transmembrane segment, and short cytoplasmic tail with a PDZ-binding motif (Sudhof, 2017; Gomez et al., 2021). The extracellular portions of α-Nrxns contain six laminin-neurexin-sex hormone-binding globulin (LNS) domains interspersed with three epidermal growth factor-like (EGF-like) domains. In contrast, β-Nrxns have a unique N-terminal histidine-rich domain (HRD) and a single LNS domain corresponding to the sixth LNS domain of α-Nrxns (LNS6) (Ushkaryov et al., 1994; Sudhof, 2017). The Nrxn1γ isoform has a very small extracellular region in which only the stalk region is conserved (Sterky et al., 2017).

Interestingly, most known Nrxn ligands interact specifically with either the αLNS6/βLNS or αLNS2 domains, highlighting these regions as critical ligand-binding interfaces (Ichtchenko et al., 1995; Missler et al., 1998; Sugita et al., 2001; Boucard et al., 2005; de Wit et al., 2009; Ko et al., 2009; Uemura et al., 2010; Zhang et al., 2010; Cheng et al., 2016). The expanded domain organization of α-Nrxns relative to β-Nrxns enables them to interact with a broader and more diverse array of ligands, underscoring their functional versatility within the synaptic cleft (Missler et al., 1998; Sugita et al., 2001; Reissner et al., 2014; Singh et al., 2016; Tanabe et al., 2017; Tong et al., 2017; Fan et al., 2021; Chofflet et al., 2024). In addition, the extended architecture of α-Nrxns, featuring multiple LNS domains connected by flexible EGF-like domain hinges, enables structural adaptability (Comoletti et al., 2010; Chen et al., 2011; Miller et al., 2011; Reissner and Missler, 2011; Tanaka et al., 2012; Reissner et al., 2013). While both α- and β-Nrxns can form trans-synaptic complexes with neuroligins (Nlgns), the larger ectodomain of α-Nrxns is expected to reduce the molecular packing density of these complexes, potentially altering the spatial organization of adhesion molecules at the synapse (Tanaka et al., 2011, 2012). As the intracellular tails of both Nrxns and their binding partners contain anchoring sites for scaffolding proteins (Hata et al., 1996; Poulopoulos et al., 2009; Jeong et al., 2019), such a sparse distribution of α-Nrxn–containing adhesion complexes would also influence cytoplasmic scaffolding dynamics. Therefore, the functional differences between α- and β-Nrxns may not arise solely from distinct ligand binding profiles but also from differences in their structural and spatial architecture, even when engaging the same synaptic partners.

Beyond differential promoter usage, vertebrate Nrxns further diversify their molecular repertoire through alternative splicing at up to six sites in α-Nrxns (SS1-SS6 for Nrxn1α and Nrxn3α; SS1-SS5 for Nrxn2α) (Ullrich et al., 1995; Schreiner et al., 2014; Treutlein et al., 2014; Gomez et al., 2021). Despite the high degree of evolutionary conservation between invertebrate and vertebrate Nrxns, extensive alternative splicing appears to be exclusive to vertebrate Nrxns (Tabuchi and Sudhof, 2002). In mammals, some of these sites contain multiple alternative donor and acceptor sequences, enabling the generation of hundreds of distinct transcript isoforms in the mouse brain (Schreiner et al., 2014; Treutlein et al., 2014). Alternative splicing patterns of Nrxns are conserved between rodents and humans, as shown by transcriptomic studies of post-mortem human brain tissue and induced pluripotent stem cell (iPSC)-derived neurons (Harkin et al., 2017; Flaherty et al., 2019). While this complex splicing program is tightly regulated in a spatial and cell-type specific manner, it remains surprisingly stable temporally during neuronal maturation (Aoto et al., 2013; Fuccillo et al., 2015; Lukacsovich et al., 2019). As some of these sites are in the ligand binding regions of Nrxns, splicing can alter the partner affinities of Nrxns: splicing at SS2 or SS4 controls the binding of Nrxn-interacting molecules to αLNS2 or αLNS6/βLNS domains (Gomez et al., 2021). For example, inclusion of the insert at SS2 in αLNS2 promotes binding to neurexophilins (Nxphs) while inhibiting interaction with dystroglycan (DAG) (Missler and Sudhof, 1998; Sugita et al., 2001; Wilson et al., 2019). Altogether, alternative splicing has evolved as a key mechanism by which vertebrate Nrxns, particulary α-Nrxns, diversify their interaction profiles to enable context-dependent regulation of synaptic connectivity.

## 3 Expression of α-Nrxns

Nrxns are broadly expressed during both embryonic and adult stages in the central nervous system (CNS) of humans and rodents, in both neuronal and glial cell types, highlighting their diverse functional roles (Ushkaryov et al., 1992; Puschel and Betz, 1995; Fuccillo et al., 2015; Furlanis et al., 2019; Uchigashima et al., 2019; Yao et al., 2021; Bose et al., 2025). In the mouse CNS, α isoforms are expressed at higher levels than β isoforms, with Nrxn2 generally showing lower expression compared to Nrxn1 and Nrxn3 (Anderson et al., 2015; Schreiner et al., 2015). In contrast, during human cortical development, NRXN1 and NRXN2 exhibit consistently higher expression levels than NRXN3 (Harkin et al., 2017). In the embryonic mouse nervous system, Nrxn1α shows widespread expression throughout the central and peripheral nervous systems, whereas Nrxn2α, and especially Nrxn3α, display more restricted regional patterns (Puschel and Betz, 1995). Notably, *Nrxn* genes are expressed as early as embryonic day 10 (E10) in mice, suggesting a role for Nrxns prior to synapse formation and maturation (Puschel and Betz, 1995). In the developing human prefrontal cortex, NRXN1α and NRXN1β are both expressed, with a pronounced increase during late embryonic and early postnatal periods, followed by a sharp decline and subsequent stabilization at around five years of age (Jenkins et al., 2016; Harkin et al., 2017). This peak in NRXN1α expression coincides with critical periods of synaptogenesis, synaptic maturation, and refinement (Andersen, 2003). In contrast, NRXN2 expression remains relatively stable in the fetal human cortex between 8 and 12 weeks post-conception. Thus, temporal dynamics in NRXN expression are isoform-specific during cortical development (Harkin et al., 2017).

Nrxns are predominantly localized to presynaptic terminals, as demonstrated by subcellular fractionation and immunogold electron microscopy (Berninghausen et al., 2007; Taniguchi et al., 2007). This presynaptic localization is further supported by their function as receptors for α-latrotoxin, a neurotoxin that induces massive neurotransmitter release by acting specifically at presynaptic sites (Ushkaryov et al., 1992). Moreover, deletion of α-Nrxns leads to pronounced impairments in neurotransmitter release and presynaptic Ca^2 +^ dynamics (Missler et al., 2003; Zhang et al., 2005; Brockhaus et al., 2018), underscoring their critical role in presynaptic function. Nonetheless, both immunogold labeling and subcellular fractionation have also identified a postsynaptic pool of Nrxns, particularly among the α isoforms (Berninghausen et al., 2007; Taniguchi et al., 2007). Additionally, immunodetection of endogenous epitope-tagged Nrxn1α in cultured cortical neurons demonstrated that, although Nrxn1α is primarily axonal, its dendritic localization progressively increases during neuronal maturation (Ribeiro et al., 2019). Supporting a postsynaptic role, α-Nrxns have been implicated in the cell-autonomous regulation of postsynaptic *N*-methyl-D-aspartate receptor (NMDAR) function (Kattenstroth et al., 2004). Differences in subcellular localization between α and β isoforms have also been observed in cultured parvalbumin-positive (PVALB) interneurons (INs), where exogenously expressed Nrxn1β is enriched at presynaptic boutons, whereas Nrxn1α is more diffusely distributed along the axon with only modest enrichment at presynaptic sites (Fu and Huang, 2010). Fluorescence recovery after photobleaching (FRAP) experiments further revealed that Nrxn1β exhibits active trafficking to and from putative synaptic sites, while Nrxn1α displays primarily passive diffusion within axonal compartments (Fu and Huang, 2010). Moreover, despite its larger extracellular domain, Nrxn1α exhibits higher surface mobility and lower synaptic confinement than Nrxn1β in cultured hippocampal neurons (Neupert et al., 2015). Despite these insights, a comprehensive understanding of the endogenous localization of Nrxn isoforms across neuronal subtypes and synapse classes remains limited, largely due to the lack of isoform-specific antibodies suitable for high-resolution mapping.

As an alternative to antibody-based experiments, epitope-tagged knock-in (KI) mouse models have been used to further describe Nrxn localization. Although studies using α-Nrxn-specific epitope-tagged KI mouse models are limited (Ribeiro et al., 2019), previous studies using epitope-tagged Nrxn1α/β and Nrxn3α/β KI lines have revealed that both isoforms form subsynaptic densities (SSDs), protein-rich synaptic microdomain regions, at glutamatergic synapses (Trotter et al., 2019; Lloyd et al., 2023). Importantly, endogenous Nrxn1 and Nrxn3 localize to discrete, non-overlapping SSDs, which are spatially aligned with distinct postsynaptic partners (Lloyd et al., 2023). This molecular compartmentalization is functionally significant as Nrxn1 is associated with the regulation of NMDA receptor (NMDAR)-mediated currents, whereas Nrxn3 selectively modulates AMPA receptor (AMPAR) strength (Aoto et al., 2013; Dai et al., 2019, 2021, 2023). Consistently, Nrxn3 forms nanocolumns with LRRTMs and AMPARs (Nozawa et al., 2022; Lloyd et al., 2023), while Nrxn1 forms nanocolumns with GluD1, Nlgns, and NMDARs (Nozawa et al., 2022; Lloyd et al., 2023). The nanoscale organization of these trans-synaptic assemblies and regulation of NMDAR and AMPAR currents is further regulated by alternative splicing of Nrxn1 and Nrxn3 at SS4, indicating a critical role for splice variant diversity in shaping synaptic architecture (Aoto et al., 2013; Dai et al., 2019; Nozawa et al., 2022). Finally, Nrxn1α is subject to proteolytic cleavage by the metalloprotease ADAM10, and pharmacological inhibition of ADAM10 significantly enhances both the presence and molecular content of Nrxn1 nanoclusters at glutamatergic synapses (Trotter et al., 2019). These findings suggest that α-Nrxns, and presumably β-Nrxns too, anterogradely control the nanoscopic arrangement of glutamate receptors in excitatory synapses by coordinating with specific postsynaptic ligands in a gene-, splice-, and post-translational modification-dependent manner.

## 4 Functions of α-Nrxns

### 4.1 Presynaptic calcium influx and neurotransmitter release

Constitutive deletion of all three *α-Nrxn* isoforms has revealed that they are required for postnatal survival: double and triple knockout (DKO and TKO) mice exhibit severe breathing deficits (Missler et al., 2003). Electrophysiological recordings in acute brainstem slices and cultured neocortical slices from newborn mice have revealed that both spontaneous and evoked GABA and glutamate neurotransmitter release are severely impaired by deletion of *α-Nrxns*, with the severity of the impairment increasing with the number of mutant alleles (Missler et al., 2003). In particular, there are selective defects in synaptic N- and P/Q-type voltage-gated Ca^2+^ channel (VGCC) activity in brainstem neurons (Missler et al., 2003; Zhang et al., 2005). Interestingly, this occurs after establishment of synaptic contacts suggesting that α-Nrxns act as synapse-specific regulators of VGCCs (Missler et al., 2003). Remarkably, transgenic expression of Nrxn1α, but not Nrxn1β, rescues evoked and spontaneous neurotransmitter release defects by improving N- and P/Q-type Ca^2+^ channel function in newborn brainstem slices from mice with various combinations of *α-Nrxn* knockout (single KO, DKO and TKO) (Missler et al., 2003; Zhang et al., 2005). These studies have highlighted two important features of Nrxns: first, as transgenic Nrxn1α can compensate for deletion of other *α-Nrxn* genes, there must be some degree of functional redundancy between the three α-Nrxns. Second, as Nrxn1β expression cannot rescue the defects, the unique extracellular domain of α-Nrxns must be responsible for regulating neurotransmitter release through modulation of VGCCs.

In cultured hippocampal neurons as well as at neuromuscular junctions, triple *α-Nrxn* deletion reduces evoked neurotransmitter release and presynaptic P/Q-type channel-mediated Ca^2+^ influx, while increasing somatic Ca^2+^ transients mediated by P/Q-channels (Sons et al., 2006; Brockhaus et al., 2018, 2024). These changes in Ca^2+^ dynamics are accompanied by a reduction in P/Q-type VGCC abundance at *α-Nrxn* TKO synapses (Brockhaus et al., 2018). While overexpression of Nrxn1α does not affect synaptic abundance of P/Q-type VGCCs and only partially restores their function, it fully normalizes presynaptic and somatic Ca^2+^ transients as well as neurotransmitter release in hippocampal neurons (Brockhaus et al., 2018). This is consistent with the ability of Nrxn1α to regulate other types of VGCCs (Zhang et al., 2005; Brockhaus et al., 2024) and to be preferentially found in the nano-environment of N-type VGCCs (Ca_*V*_2.2) in the mouse brain (Muller et al., 2010). In combination with the α2δ-1, but not the α2δ-3, Ca_*V*_ auxiliary subunit, Nrxn1α facilitates Ca^2+^ presynaptic influx and enhances currents through P/Q-type channels (Brockhaus et al., 2018). This effect appears specific to the cooperation between Nrxn1α and α2δ-1 and likely involves the modulation of the surface presence of activable channels, rather than changes in the kinetic properties of P/Q-type channels (Missler et al., 2003; Dudanova et al., 2006; Brockhaus et al., 2018). In this context, Nrxn1α does not appear to form a stable complex with α2δ-1 or α2δ-3 proteins (Brockhaus et al., 2018), in contrast to another report (Tong et al., 2017). Instead, α-Nrxns differentially modulate the surface mobility of α2δ-1 and α2δ-3 in neurons, supporting the idea that regulation of presynaptic Ca^2+^ transients (PreCaTs) is a highly dynamic process that is sensitive to transient protein associations (Schneider et al., 2015; Brockhaus et al., 2018).

A recent study found that single deletion of *Nrxn1α* results in a decreased contribution of L-type channels to PreCaTs, while N-type channel contribution increases, with no effect on P/Q-type channels in cultured hippocampal neurons (Brockhaus et al., 2024). In contrast, pan-*Nrxn* KO (α and β isoforms) primarily reduces P/Q-type-mediated PreCaTs, accompanied by a relative increase in the contribution of L- and R-type VGCCs (Brockhaus et al., 2024). Consistently, pan-*Nrxn* deletion leads to a reduction in P/Q-channels abundance and impairs coupling between PreCaTs and neurotransmitter release at calyx of Held synapses (Luo et al., 2020). While overexpression of Nrxn1α ameliorates several synaptic defects observed in *α-Nrxn1/2* DKO at excitatory and inhibitory synapses of mouse newborn brainstems, including a partial rescue of P/Q-type channel dysfunctions, these findings suggest that individual Nrxn isoforms support synaptic transmission through distinct types of presynaptic VGCCs (Zhang et al., 2005). Furthermore, regulation of VGCCs by Nrxns is likely to be highly context-dependent, given that the expression and clustering of VGCC subtypes vary at different types of synapses and during development (Iwasaki et al., 2000; Sons et al., 2006; Nakamura et al., 2015).

Inhibition of neurotransmitter release by endocannabinoid signaling is mediated by modulation of presynaptic VGCCs and a reduction in PreCaTs (Brown et al., 2004). *β-Nrxn* TKO reduces both evoked and spontaneous glutamatergic synaptic transmission, as well as PreCaTs, without altering the synaptic abundance of VGCCs in cultured cortical and hippocampal neurons (Anderson et al., 2015; Klatt et al., 2021). These defects in basal in synaptic transmission can be rescued by overexpression of Nrxn1β, but not Nrxn1α, in cortical neurons, or by pharmacological inhibition of the endocannabinoid pathway, suggesting that β-Nrxns specifically regulate tonic endocannabinoid-mediated neurotransmitter release (Anderson et al., 2015). However, while the reduction of PreCaTs by cannabinoid receptor 1 (CB1R) activation by 2-arachidonoylglycerol (2-AG) was dampened by 25% in pan-*Nrxn* KO hippocampal neurons, it was only diminished by 5% in *β-Nrxn* TKO neurons, indicating a potential role for α-Nrxns in tonic endocannabinoid-mediated synaptic inhibition (Brockhaus et al., 2024). Nevertheless, the inability of Nrxn1α overexpression to rescue the phenotypes observed in *β-Nrxn* TKO cortical neurons suggests that α- and β-Nrxns may regulate endocannabinoid-mediated neurotransmission via non-overlapping mechanisms in a context-dependent manner. Interestingly, presynaptic defects in *β-Nrxn* TKO cortical neurons appear to be caused by elevated 2-AG- but not anandamide (AEA)-mediated tonic endocannabinoid signaling. In contrast, loss of *Nrxn1α* results in reduced release probability caused by endocannabinoid dysregulation through the AEA, but not the 2-AG, pathway at corticostriatal synapses in acute brain slices (Davatolhagh and Fuccillo, 2021). Altogether, these studies suggest that, in addition to β-Nrxns, α-Nrxns also contribute to the regulation of retrograde tonic endocannabinoid signaling, thereby modulating baseline neurotransmitter release in a context-dependent manner. Furthermore, the potential role of Nrxns in controlling phasic endocannabinoid signaling remains to be formally investigated in future studies.

Aside from their roles in modulating VGCC function in the context of synaptic transmission, α-Nrxns also control two types of Ca^2+^-dependent endocrine secretion: (1) from pituitary melanotrophs (Dudanova et al., 2006; Mosedale et al., 2012) and (2) from pancreatic β-cells (Dudanova et al., 2006; Mosedale et al., 2012). Adult DKO mice lacking either *Nrxn1α/2α* or *Nrxn2α/3α* exhibit smaller body weights, atrophied pituitary glands with smaller melanotrophs and an almost complete inability to breed (Dudanova et al., 2006). Patch-clamp measurements have revealed reduced Ca^2+^-dependent secretory activity of *α-Nrxn1/2* or *2/3* DKO adult mouse melanotrophs without ultrastructural changes of secretory granules (Dudanova et al., 2006). Interestingly, *α-Nrxn1/2* or *2/3* DKO as well as TKO newborn mice also exhibit impaired melanotroph secretory activity but without differences in the size of the pituitary lobes or in cell size, suggesting that a functional rather than morphological defect is the primary phenotype in the pituitary gland (Dudanova et al., 2006). Unlike in brainstem and neocortical neurons (Missler et al., 2003; Zhang et al., 2005), *α-Nrxn* deletion (*α-Nrxn1/2* or *2/3* DKO or TKO) does not lead to a reduction in Ca^2+^ currents but instead results in a small shift in the voltage dependence of Ca^2+^ currents in melanotrophs (Dudanova et al., 2006). Thus, it was proposed that α-Nrxns are crucial for the coupling of VGCCs to release-ready vesicles and metabotropic GABA_*B*_R in melanotrophs, as is also the case in newborn brainstem neurons and at calyx of Held synapses (Dudanova et al., 2006; Luo et al., 2021). Although pituitary melanotrophs express GABA_*B*_R (Purisai et al., 2005), it remains to be demonstrated that the secretory defects observed in *α-Nrxn* KO mice are specifically due to the loss of GABA_*B*_R modulation of VDCCs in melanotrophs. Additionally, *α-Nrxn1/2* and *2/3* DKO adult mice exhibit a profound reduction in Ca^2+^-dependent synaptic transmission in the hypothalamo-hypophysial axis, but this defect cannot explain the reduced secretory activity of melanotrophs since newborn mice lack these hypothalamic inputs (Dudanova et al., 2006). In support of a role for α-Nrxns in the second type of Ca^2+^-dependent endocrine secretion, deletion of *Nrxn1α* results in increased glucose-stimulated but not basal insulin secretion from pancreatic islets (Mosedale et al., 2012). While the percentage of plasma membrane-docked insulin granules is reduced in *Nrxn1α* KO islets, the total number of granules per cell is increased (Mosedale et al., 2012). Altogether, α-Nrxns and their ligands are expressed in different endocrine systems, and their genetic deletion support the idea of a generalized function in Ca^2+^-dependent endocrine secretion (Dudanova et al., 2006; Suckow et al., 2008; Mosedale et al., 2012; Suckow et al., 2012; Shah et al., 2023). Emerging evidence suggests a link between neurodevelopmental as well as mental disorders and metabolic syndromes, highlighting the possibility that dysfunction in *α-NRXNs* may contribute not only to neuronal impairment but also to systemic physiological disturbances (Farooqui et al., 2012; Penninx and Lange, 2018; Liu S. et al., 2021).

### 4.2 Regulation of NMDAR functions

*β-Nrxn* TKO cultured cortical neurons exhibit a reduction in both NMDAR- and AMPAR-mediated evoked EPSCs caused by a diminution in release probability (demonstrated by slower use-dependent blockade of evoked NMDAR-EPSCs by the NMDAR antagonist MK-801; (Anderson et al., 2015). In contrast, neocortical slices from *α-Nrxn* TKO mice show a profound reduction in NMDAR- but not AMPAR-mediated spontaneous and evoked EPSCs (Kattenstroth et al., 2004). This selective impairment suggests that α-Nrxns directly regulate NMDAR functions, but the exact mechanisms remain unclear. In *Nrxn1α* KO mice, NMDAR currents are reduced at thalamostriatal but not at thalamocortical or hippocampal synapses (Etherton et al., 2009; Davatolhagh and Fuccillo, 2021), whereas a robust increase and decrease in NMDAR- and AMPAR-mediated currents, respectively, occurs in corticoamygdalar pathways (Asede et al., 2020). Deletion of *Nrxn2α* selectively impairs NMDAR-mediated synaptic transmission at somatosensory cortical synapses, as evidenced by a reduction in NMDAR-EPSC amplitude and shortened decay time - consistent with a loss of slow-kinetic NMDAR function (Traynelis et al., 2010; Born et al., 2015). Bath application of the NMDAR antagonist APV or intracellular delivery of MK-801 reduces EPSC decay time in wild-type (WT) neurons, but has no effect in *Nrxn2α* KO neurons, suggesting deficits in postsynaptic NMDARs (Born et al., 2015). In addition, *Nrxn2α* deletion leads to a reduction in paired-pulse facilitation (PPF) with, atypically, an associated decreased presynaptic release probability (Born et al., 2015). Notably, this change in short-term plasticity appears to be NMDAR-dependent, as APV application reduces PPF in WT but not in *Nrxn2α* KO neurons (Born et al., 2015), consistent with a role for NMDARs in modulating PPF (Zinebi et al., 2001; Akopian and Walsh, 2002). Interestingly, additional deletion of *Nrxn2β* does not significantly exacerbate these phenotypes despite widespread Nrxn2β expression in neocortical regions (Born et al., 2015; Uchigashima et al., 2019). These findings highlight Nrxn2α as the principal Nrxn2 isoform responsible for the regulation of NMDAR functions. Importantly, the NMDAR dysfunction observed in *α-Nrxn* TKO or *Nrxn2α* single KO neurons is unlikely to be caused by dysregulation of the alternative splicing-dependent regulation of AMPAR and NMDAR responses by Nrxns (Aoto et al., 2013; Dai et al., 2019). Constitutive insertion at SS4 in any Nrxn isoform increases NMDAR-mediated currents, while constitutive removal of the SS4 insert does not alter NMDAR function in hippocampal neurons (Dai et al., 2019). Additionally, alternative splicing of Nrxn2 at SS4 has no effect on NMDAR-mediated synaptic responses (Dai et al., 2019). Interestingly, postsynaptic NMDAR-mediated synaptic transmission and PPF are regulated by α2δ-1 and L-type VGCCs (Akopian and Walsh, 2002; Chen et al., 2018). Given that α-Nrxns regulate α2δ-1 surface mobility (Brockhaus et al., 2018), as well as L-type Ca^2+^ channel function (Brockhaus et al., 2024), future studies assessing the role of VGCCs in α-Nrxn-mediated modulation of NMDARs would be valuable.

### 4.3 Regulation of GABAergic inhibitory transmission

As previously discussed, triple *α-Nrxn* deletion results in profound reduction of spontaneous and evoked GABAergic transmission in the brainstem and neocortical regions. These impairments are caused by dysfunctions in VGCCs and by a reduction in inhibitory synapse density (Missler et al., 2003; Zhang et al., 2005; Dudanova et al., 2007) likely due to impaired assembly as α-Nrxns have been shown to interact with and to recruit inhibitory postsynaptic components such as GABA_*A*_R or Nlgn2 (Kang et al., 2008; Geisler et al., 2019; Miyazaki et al., 2021).

In contrast to the triple KOs, perturbations of individual α-Nrxn isoforms typically produce modest and context-dependent effects on inhibitory synapses. Deletion of *Nrxn1α* or *Nrxn2α* does not alter spontaneous global GABAergic transmission or inhibitory synapse density in hippocampal or cortical acute brains slices (Etherton et al., 2009; Born et al., 2015). However, a specific trans-synaptic complex composed of Nrxn1α-Nlgn3 that is critically dependent on alternative splicing of Nlgn3 (lacking both A1 and A2 inserts) and Nrxn1α (requiring the SS4 insert) regulates synaptic strength between cholecystokinin (CCK)-expressing INs and pyramidal neurons (PN) (Uchigashima et al., 2020). In the basal amygdala (BA), loss of *Nrxn1α* impairs local inhibition by decreasing inhibitory connectivity and reducing GABAergic synaptic tone (Asede et al., 2020). These defects impair feedforward inhibition from both the dorsomedial prefrontal cortex and lateral amygdala to BA pathways (Asede et al., 2020). Notably, these functional impairments are not caused by a reduction in perisomatic inhibitory synapse density (Asede et al., 2020).

These findings suggest that individual α-Nrxn isoforms control GABAergic synaptic transmission in a cell type-specific manner, and that concurrent perturbation of multiple isoforms results in cumulative phenotypic effects on synaptic inhibition. Supporting this notion, in the ventral subiculum (vSUB), Nrxn3 controls synaptic inhibition mediated by PVALB-expressing INs onto regular-spiking (RS) PNs in a sex-dependent manner (Boxer et al., 2021). In males, Nrxn3 controls the density and strength of PVALB-RS synapses, whereas in females, it regulates their presynaptic release probability (Boxer et al., 2021). Notably, Nrxn3 appears to be dispensable for PVALB IN synapses onto burst-firing (BS) PNs (Boxer et al., 2021). Despite these functional differences, the level of Nrxn3α, the primary isoform expressed in vSUB PVALB + INs, is comparable between sexes, suggesting that the observed sexual dimorphism arises downstream or via interacting factors (Boxer et al., 2021). Additionally, Nrxn3α is essential for normal global GABAergic transmission in olfactory bulb and mPFC neurons, but not in hippocampal neurons (Aoto et al., 2015; Trotter et al., 2023). Furthermore, Nrxn3α mediates trans-synaptic signaling through DAG to regulate inhibitory synapse function, a process that is modulated by alternative splicing at SS2 (Trotter et al., 2023).

These findings underscore the highly context-dependent roles of α-Nrxns in regulating GABAergic synaptic function, shaped by factors such as cell type, brain region, and alternative splicing; importantly, emerging evidence points to biological sex as an additional layer of regulation, warranting further investigation in future studies. Furthermore, multiple deletion of *α-Nrxns* leads to a selective reduction in inhibitory synapse density while largely preserving excitatory synapse density and synaptic ultrastructure (Missler et al., 2003; Dudanova et al., 2007). It remains unclear whether α-Nrxns also play a role in the early formation of inhibitory synapses or if these synapses are uniquely vulnerable to functional impairments.

### 4.4 Modest nervous system morphological defects caused by deletion of α-Nrxns

Despite widespread temporospatial expression throughout the CNS, deletion of *α-Nrxns* results in only modest morphological changes in the CNS. Early studies using *α-Nrxn* DKO and TKO mice reported no major structural brain abnormalities, axon pathfinding defects, or increases in apoptosis (Missler et al., 2003; Dudanova et al., 2007). However, more subtle changes have been observed: in newborn TKO mice, olfactory glomeruli are reduced by approximately 20%, and in adult DKO mice, there is a similar 20% reduction in neuropil area along with a shortening of distal dendritic branches (Dudanova et al., 2007).

At the ultrastructural level, both asymmetric and symmetric synapses have normal morphology in *α-Nrxn* DKO and TKO mice (Missler et al., 2003). While the density of symmetric (inhibitory) synapses is selectively reduced by approximately 30%–40% in neocortical and brainstem areas of newborn TKO and adult DKO mice, asymmetric (excitatory) synapses remain unaffected (Missler et al., 2003; Dudanova et al., 2007). Whether the decrease in inhibitory synapse density reflects impaired early synapse formation or defective maintenance remains unclear. Future studies using conditional *α-Nrxn* deletion in mice at adult stages are needed to address this question.

While the somatosensory cortex shows no changes in the density or ultrastructure of inhibitory or excitatory synapses (Born et al., 2015), diffusion tensor imaging reveals altered brain microstructure in *Nrxn2α* KO mice (Pervolaraki et al., 2019). These mice exhibit increased fractional anisotropy (FA) in the amygdala, orbitofrontal cortex (OFC), anterior cingulate cortex (ACC), and hippocampus, alongside reduced FA in the basolateral amygdala (BLA). Disruptions in axonal fiber integrity are also observed in the amygdala, OFC, and ACC. Thus, *Nrxn2α* KO mice have atypical structural connectivity across brain regions implicated in social behavior and anxiety (Pervolaraki et al., 2019).

In the peripheral nervous system, *α-Nrxn* DKO causes only modest structural abnormalities. At the neuromuscular junction, synaptic morphology appears largely preserved, and overall synapse density remains unchanged in DKO mice (Sons et al., 2006).

Overall, deletion of *α-Nrxns* results in modest and specific morphological changes in both the central and the peripheral nervous system. Despite severe defects in neurotransmitter release at both excitatory and inhibitory synapses throughout the CNS, *α-Nrxn1/2* or *2/3* DKO and TKO mice exhibit a selective reduction in inhibitory synapses. This suggests that α-Nrxns act primarily as functional regulators of synaptic transmission rather than as building blocks for nerve connections. Notably, phenotypic severity correlates with the number of mutant *α-Nrxn* alleles, suggesting a dosage-dependent effect and potential functional redundancy among α-Nrxn isoforms.

## 5 α-Nrxn specific ligands

**FIGURE 1:**
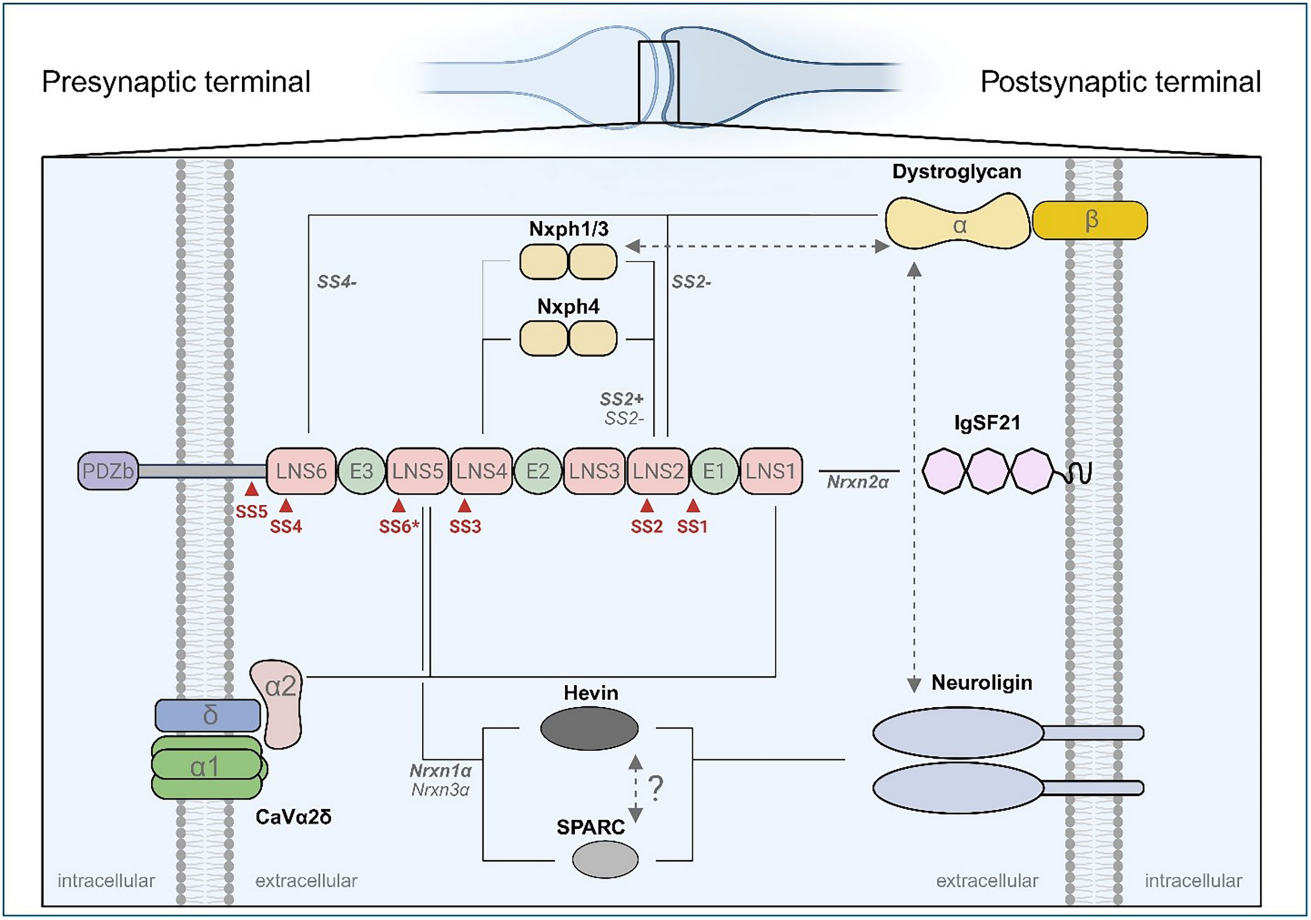
Extracellular architecture of presynaptic α-neurexins and their binding partners across the synaptic cleft. Schematic representation of α-neurexin (α-Nrxn) interactions with specific ligands in the synaptic context. The domain organization of presynaptic α-Nrxns is shown, comprising six Laminin-Neurexin-Sex hormone-binding globulin (LNS1–6) domains interspersed by three epidermal growth factor-like (EGF1–3) domains, and terminating with a cytoplasmic PDZ-binding motif (PDZb). Red triangles indicate alternative splice sites (SS1–SS6), with SS6* absent in *Nrxn2*α. Solid lines represent known protein–protein interactions, while dashed lines with double arrowheads indicate competitive binding between ligands. The gray solid line connecting Nxph1/3 to LNS4 represents a putative interaction, based on the demonstrated binding of Nxph4 to LNS4. The competitive relationship between hevin and SPARC is also depicted, though this interaction remains to be experimentally confirmed. Where known, ligand specificity for neurexin splice variants and gene isoforms is noted in bold italics. The diagram provides spatial orientation across the synaptic cleft, with presynaptic and postsynaptic compartments and associated molecules indicated. Proteins and structures are not drawn to scale.

Although many Nrxn ligands bind to both α-Nrxns and β-Nrxns (Sudhof, 2017), a few Nrxn-binding proteins have been isolated as α-Nrxn-specific ligands, for example, Nxphs (Missler et al., 1998), immunoglobulin superfamily member 21 (IgSF21) (Tanabe et al., 2017), high endothelial venule protein (hevin) (Singh et al., 2016) and voltage-gated calcium channel auxiliary subunit α2δ (CaVα2δ) (Tong et al., 2017; Figure 1). Furthermore, DAG was isolated as a α- and β-Nrxn-binding protein (Sugita et al., 2001; Reissner et al., 2014) but appears to regulate inhibitory synapse function through α-Nrxns. Each of these α-Nrxn ligands is discussed in detail here.

### 5.1 Neurexophilins

Nxphs are small, secreted glycoproteins that were first purified in complex with Nrxn1α on immobilized alpha-latrotoxin (Petrenko et al., 1996; Missler and Sudhof, 1998). Nxphs are expressed by four genes in mammals (Nxph1-4) but absent in invertebrates (Missler and Sudhof, 1998). Structurally, Nxphs are composed of a variable N-terminal pro-domain, a conserved C-terminal mature fragment composed of an N-glycosylated domain, and a C-terminal cysteine-rich domain (Missler et al., 1998; Missler and Sudhof, 1998). Nxphs are expressed as N-glycosylated preproteins in all cell types but only undergo proteolytic cleavage in neuronal cells (Missler et al., 1998; Missler and Sudhof, 1998). While initially only Nxph1 and 3 were shown to bind α-Nrxns through their second LNS domain (Missler et al., 1998), a subsequent study demonstrated that Nxph4 acts as a ligand of α-Nrxns *in vivo* (Meng et al., 2019). Interestingly, Nxph4 co-immunoprecipitates with single LNS2 and LNS4 domains suggesting that α-Nrxns contain two distinct Nxph-binding sites (Meng et al., 2019). While N-glycosylation of Nxph1 is not a prerequisite for its interaction with Nrxn1α, it stabilizes the Nxph1-Nrxn1α complex (Reissner et al., 2014). Analysis of the crystal structure of the Nxph1 mature C-terminal fragment in complex with the Nrxn1α LNS2 domain revealed that both proteins form a large contiguous beta-sandwich by alignment of their individual beta-sandwiches and that insertion at Nrxn1α splicing site 2 (SS2) strengthens the interaction with Nxph1 by addition of specific contacts sites at the Nxph1-LNS2 interface as well as by stabilizing nearby hydrophobic interactions (Wilson et al., 2019).

Nxphs isoforms exhibit distinct expression patterns in the brain. Rodents express Nxph1, 2 and 4, while humans express NXPH2, 3 and 4 (Missler and Sudhof, 1998). Nxph1 is present in scattered neurons across the adult rat brain, in a pattern suggestive of expression in inhibitory INs (Petrenko et al., 1996). Notably, in the hippocampus, Nxph1 mRNA is absent from PNs and granule cells while it is present at high levels in dispersed cells that appear to be inhibitory INs (Petrenko et al., 1996). In the olfactory bulb, Nxph1 is uniformly expressed by inhibitory periglomerular neurons and is found in glutamatergic tufted cells. Strong Nxph1 expression is also present in some thalamic nuclei (Petrenko et al., 1996). At the ultrastructural level, immunogold labeling revealed that, while Nrxns are present in neocortical asymmetric and symmetric synapses, Nxph1 is exclusively present at symmetric inhibitory synapses (Reissner et al., 2014). In contrast to the dispersed expression of Nxph1, Nxph3 and Nxph4 expression is much more restricted. Nxph3 is enriched in non-GABAergic layer 6b cortical cells as well as granule cells in lobules 9 and 10 of the cerebellar vermis in the adult mouse brain (Beglopoulos et al., 2005) and expressed by glutamatergic neurons of the deep cerebellar nuclei as well as inhibitory Golgi cells in the cerebellar cortex (Meng et al., 2019). Nxph4 is expressed in specific interconnected brain regions and cell types relevant for motor control, food and energy balance, and olfactory and emotional function (Meng et al., 2019).

Functional studies, including both loss- and gain-of-function (LOF and GOF) approaches, have implicated Nxphs in the regulation of inhibitory synaptic transmission in the brain. *Nxph1* KO mice show no differences in mortality or body weight (Beglopoulos et al., 2005), but electrophysiological recordings reveal an increased frequency of miniature inhibitory postsynaptic currents (mIPSCs) in thalamic reticular nucleus inhibitory neurons as well as impaired GABA_*B*_R-dependent short-term presynaptic depression at inhibitory synapses (Born et al., 2014). Interestingly, ectopic expression of Nxph1 at cortical excitatory synapses results in hindered GABA_*B*_R- and GABA_*A*_R-dependent short-term presynaptic facilitation (Born et al., 2014). These alterations are accompanied by increased GABA_*B*_R and GABA_*A*_R expression at excitatory synapses, suggesting that, similar to α-Nrxns, Nxph1 has an instructive role at synapses (Born et al., 2014; Miyazaki et al., 2021). Consistent with its function at inhibitory synapses, Nxph1 is a major endogenous interacting partner of Nrxn3 SS5+, an Nrxn3 isoform selectively involved in dendritic inhibition (Hauser et al., 2022). As is the case for *Nxph1*, deletion of *Nxph3* results in no difference in mortality or body weight, and this persists even when combined with *Nxph1* deletion (Beglopoulos et al., 2005). *Nxph3* KO mice do not exhibit any gross brain morphological abnormalities, but do display impaired sensorimotor gating as well as motor coordination defects (Beglopoulos et al., 2005). In contrast, global homozygous deletion of *Nxph4* results in lower body weight, motor coordination defects and reduced anxiety in mice of both sexes as well as female-specific sensorimotor gating deficits (Meng et al., 2019). In the cerebellum, *Nxph4* KO mice exhibit drastically reduced GABAergic inhibition between Golgi and granule cells with a modest reduction in GABAergic synapses density, but glutamatergic synaptic transmission between mossy fibers and granule cells remains unaltered (Meng et al., 2019). Interestingly, Nxph4 interacts with GABA_*A*_R in cerebellar synaptosomes, suggesting a model in which α-Nrxns-Nxph4-GABA_*A*_R tripartite assembly controls GABAergic connectivity between Golgi and granule cells (Meng et al., 2019). Altogether, α-Nrxn-interacting Nxphs are expressed in non-overlapping patterns across rodent brains where they appear to selectively control GABAergic transmission.

### 5.2 High endothelial venule protein (hevin)/secreted protein acidic and rich in cysteine-like 1 (SPARCL1)

Hevin, also known as SPARCL1, and its close homolog SPARC, are secreted, glycosylated matricellular proteins with collagen-binding ability (Johnston et al., 1990; Yan and Sage, 1999; Hambrock et al., 2003; Eroglu, 2009; Jones and Bouvier, 2014; Yuzaki, 2018; Fan et al., 2021). Accumulating evidence links hevin and SPARC to autism spectrum disorders (ASD) (De Rubeis et al., 2014; Wallingford et al., 2017; Taketomi et al., 2022; Taketomi and Tsuruta, 2023), alcohol use disorder (Nunez-delMoral et al., 2023), and chronic pain (Chen et al., 2022; Kang et al., 2022) as well as Alzheimer’s disease (Jayakumar et al., 2017; Seddighi et al., 2018; Strunz et al., 2019; Cabral-Miranda et al., 2025).

During embryonic development, hevin and SPARC are expressed in radial glia as well as in the developing vasculature. Postnatally, both proteins are heavily secreted by astrocytes with hevin also expressed in neurons (Lively and Brown, 2008; Eroglu, 2009; Kucukdereli et al., 2011; Jones and Bouvier, 2014; Mongredien et al., 2019), while SPARC expression is restricted to glial cells (Mendis et al., 1995; Bampton et al., 2005; Cahoy et al., 2008; Vincent et al., 2008). Hevin was initially identified as a glycoprotein found in rat brain synaptosomes and named synaptic cleft protein 1 (SC1) (Johnston et al., 1990). Subsequent studies confirmed that hevin is found at excitatory synapses, notably thalamocortical synapses (Kucukdereli et al., 2011), as well as at perisynaptic glial processes (Lively et al., 2007; Lively and Brown, 2008). Interestingly, hevin and SPARC expression are developmentally regulated, reaching a peak between postnatal 15 and 25 days, which corresponds to the synaptogenesis surge (Risher et al., 2014; Singh et al., 2016). However, while SPARC expression is rapidly downregulated, hevin expression remains stable in adulthood (Kucukdereli et al., 2011). Consistent with a role in nerve repair and synaptic reorganization, hevin and SPARC expression are upregulated following CNS injury during reactive gliosis (McKinnon and Margolskee, 1996; Mendis et al., 1996, 2000; Liu et al., 2005; Lively and Brown, 2007).

Structurally, hevin and SPARC contain a flexible acidic N-terminal region followed by a globular C-terminal region encompassing a follistatin-like (FS) domain and an extracellular calcium-binding (EC) domain (Hohenester et al., 1997; Yan and Sage, 1999). While hevin and SPARC have unique N-terminal acidic domains, they share about 61% sequence identity in their FS-EC tandem region (Bradshaw, 2012). In the brain, hevin undergoes proteolytic cleavage by ADAMTS4 and MMP-3, resulting in the production of a SPARC-like fragment (SLF) containing the FS-EC region (Weaver et al., 2010, 2011; Nunez-delMoral et al., 2021). Despite their relatively high sequence homology, the 3D structures of the hevin and SPARC FS-EC regions are fundamentally different due to distinct organization of the EC domain (Fan et al., 2021). The helices linking the FS-EC tandem drive the FS domain away from the EC domain in hevin, leading to an “open” conformation (Fan et al., 2021). The C-terminal region of hevin binds the LNS5 domain of Nrxn1α, with the FS domain strengthening the interaction (Singh et al., 2016; Fan et al., 2021). Hevin also interacts weakly with Nrxn3α but fails to bind to Nrxn2α (Singh et al., 2016). Interestingly, hevin bridges Nrxn1α and Nlgn1 (B +), two proteins that otherwise do not directly interact, by simultaneously binding to both and facilitating trans-synaptic adhesion to promote synapse formation (Singh et al., 2016).

While both hevin and SPARC bind to Nrxn1α and Nlgns, it is currently not known whether they compete for these interactions (Fan et al., 2021). In cultured retinal ganglion cells (RGCs), astrocyte-secreted hevin promotes the formation of glutamatergic synapses, but, while these synapses are ultrastructurally normal, they are functionally silent (Kucukdereli et al., 2011). SPARC and the C-terminal SLF fragment of hevin antagonize hevin’s synaptogenic activity in a dose-dependent manner (Kucukdereli et al., 2011). Interestingly, hevin, SPARC and the SLF all promote neurite outgrowth and branching, suggesting that the domain and/or receptor requirements for hevin-mediated synapse formation are distinct from those for neurite outgrowth (Kucukdereli et al., 2011). *In vivo*, hevin and SPARC have opposite effects on retinocollicular synaptogenesis: synapse density decreases in *hevin* KO mice but increases in *SPARC* KO mice (Kucukdereli et al., 2011). Subsequent studies have shown that hevin additionally controls the formation, maturation, and maintenance of thalamocortical, but not intracortical, synapses (Risher et al., 2014; Singh et al., 2016). In cultured RGCs, hevin induces pre- and postsynaptic differentiation through Nrxn1α and Nlgns, respectively (Singh et al., 2016). Consistent with previous observations, hevin induces the formation of presynaptically active and postsynaptically silent synapses (NMDAR-positive but AMPAR-negative synapses) (Singh et al., 2016). From these studies, an elegant model emerged in which astrocyte-secreted hevin bridges presynaptic Nrxn1α and postsynaptic Nlgn1 (B+) to promote thalamocortical synapse formation (Singh et al., 2016). Importantly, the synaptogenic activity of hevin is abolished by deletion of either *Nrxn1α* or *Nlgn1* in co-cultured thalamic and cortical neurons. However, subsequent studies have shown that, in cultured mouse cortical neurons, hevin induces active excitatory synapse formation in a Nrxn- and Nlgn-independent manner (Gan and Sudhof, 2020), and failed to observe any interaction between hevin and Nlgn1 (Elegheert et al., 2017). Additionally, in human embryonic stem cell (hESC)-derived glutamatergic neurons, young mouse serum-derived hevin induces the formation of functional excitatory synapses with recruitment of both postsynaptic AMPARs and NMDARs (Gan and Sudhof, 2019). While it is currently challenging to reconcile these observations, it is possible that these dissimilarities originate from the different experimental models used to probe hevin’s functions. For example, the observation that hevin selectively induces the formation of Nrxn1α- and Nlgn1-dependent thalamocortical, but not intracortical, synapses was made *in vivo* (Singh et al., 2016). It is possible that this selectivity is lost in two-dimensional cultures and that other receptors mediate hevin’s synaptogenic activity at intracortical synapses (Singh et al., 2016; Gan and Sudhof, 2020). Despite the aforementioned discrepancies, LOF and GOF studies have consistently reported that hevin selectively controls glutamatergic, but not GABAergic, synapse formation (Kucukdereli et al., 2011; Gan and Sudhof, 2020).

Apart from its role in promoting synapse formation, astrocyte-secreted hevin is required for ocular dominance plasticity following monocular deprivation (Singh et al., 2016) and morphological plasticity of dendritic spines upon exposure to enriched environmental experiences (Le et al., 2025). Additionally, hevin is required for appropriate termination of radial glia-guided neuronal migration and normal cortical lamination (Gongidi et al., 2004). Furthermore, astrocyte-released hevin is involved in synaptic reorganization and nerve repair following injury (Ge et al., 2019; Brayman et al., 2021; Kim et al., 2021; Yamagata, 2021). Although *hevin* KO mice are viable, fertile and without any obvious histological or basal nociception abnormalities (McKinnon et al., 2000; Kang et al., 2011; Chen et al., 2022), future studies should be conducted to probe the involvement of Nrxn1α and Nlgns in hevin-mediated plasticity and synaptic reorganization and assess in-depth brain development and behavioral abnormalities in *hevin* KO mice.

### 5.3 Immunoglobulin superfamily member 21 (IgSF21)

IgSF21 was isolated as a postsynaptic adhesion molecule that selectively induces presynaptic GABAergic differentiation through interacting with axonal Nrxn2α (Tanabe et al., 2017; Chofflet et al., 2024). IgSF21 is a glycosylphosphatidylinositol (GPI)-anchored protein containing either two or three Ig domains depending on alternative spicing (long and short IgSF21 isoforms) that is primarily expressed in PNs (Tanabe et al., 2017; Chofflet et al., 2024). Global deletion of *IgSF21* in mice selectively impairs GABAergic synapse organization and function in both the hippocampus and the cortex (Tanabe et al., 2017). Notably, IgSF21 preferentially regulates dendrite-targeted inhibitory synapse organization. In agreement with *in vivo* loss of function studies, IgSF21 neuronal overexpression increases dendritic, but not somatic, VGAT immunoreactivity (Chofflet et al., 2024). On the other hand, Nlgn2 overexpression promotes VGAT clustering along the somatodendritic axis of transfected neurons. Interestingly, IgSF21- and Nlgn2-mediated GABAergic presynaptic differentiation relies on only partially overlapping signaling pathways in cultured hippocampal neurons. Pharmacological inhibition of JNK, CaMKII and Src signaling pathways suppresses Nlgn2-mediated induction of inhibitory presynaptic differentiation, while the synaptogenic activity of IgSF21 is only sensitive to JNK signaling blockade (Chofflet et al., 2024). Whether intracellular signaling pathways participate in the dendritic selectivity of the regulation of inhibition by IgSF21 remains to be determined.

*In silico* predictions and site-directed mutagenesis revealed that the first Ig domain of IgSF21 interacts with the first LNS domain of Nrxn2α through a hydrogen bonding network (Chofflet et al., 2024). Interestingly, the LNS1 domain of α-Nrxns is poorly conserved between the three genes, possibly explaining why IgSF21 binds to Nrxn2α, but not to Nrxn1α or Nrxn3α. Although alternative splicing of IgSF21 does not regulate its affinity for Nrxn2α, the long isoform of IgSF21 induces stronger GABAergic presynaptic differentiation than the short isoform (Tanabe et al., 2017). Interestingly, the ratio of long to short IgSF21 is higher in the mouse brain at P14, corresponding to the peak time period of GABAergic synaptogenesis. Thus, the synaptic IgSF21-Nrxn2α complex is emerging as a selective regulator of dendritic inhibition in the brain.

### 5.4 Voltage-gated calcium channel auxiliary subunit α2δ (CaVα2δ)

Alpha 2 delta (α2δ) proteins are auxiliary subunits of voltage-gated calcium channels (CaVs) encoded by four genes in mammals (*CACNA2D1-4*) and 2 genes in *Caenorhabditis elegans* (*unc-36* and *tag-180*). In humans, α2δ subunits appear to be risk genes for ASD (Iossifov et al., 2012; De Rubeis et al., 2014) and schizophrenia (Purcell et al., 2014; Moons et al., 2016), and are also linked to other neurological disorders (Pippucci et al., 2013; Valence et al., 2019). α2δ subunits regulate CaV trafficking on the plasma membrane and to the active zone to promote the coupling of CaV function to synaptic vesicle exocytosis and neurotransmitter release (Canti et al., 2005; Bernstein and Jones, 2007; Hoppa et al., 2012; Fell et al., 2016). They also modulate CaV channel function by shifting voltage-dependent activation, altering steady-state inactivation, and accelerating inactivation (Felix et al., 1997; Klugbauer et al., 1999). In cholinergic neuromuscular junctions in *Caenorhabditis elegans*, the ectodomain cleaved from postsynaptic NRX-1α participates in retrograde synaptic inhibition by suppresing neurotransmitter release coupled to UNC2-CaV2s through binding to UNC-36/α2δ (Tong et al., 2017). Additionally, postsynaptic NRX-1α modulates presynaptic localization of UNC-36/α2δ, and presynaptic NLG-1 is required for NRX-1α-mediated retrograde synaptic inhibition (Tong et al., 2017). Interestingly, mouse Nrxn1α can form a complex with any rodent α2δ protein (α2δ-1, -2 or -3) in HEK293T cells, with α2δ-3 having the highest affinity for mouse Nrxn1α. Remarkably, mouse Nrxn1α decreases current density of CaV2.2s in an α2δ-3-dependent manner in a human cell line. When co-expressed with CaV2.2s containing either α2δ-1 or α2δ-2, Nrxn1α has no effect on calcium currents. Although it remains unknown whether α-Nrxns participate in retrograde synaptic inhibition through α2δ-3-containing CaV2.2s in mammals, the LNS1 and LNS5 domains of Nrxn1α are responsible for binding to α2δ-3 and decreasing CaV2.2 currents, arguing for an α-Nrxn-specific function.

Interestingly, another study reported that triple *α-Nrxn* KO hippocampal neurons display reduced CaV2.1-mediated presynaptic Ca^2+^ influx as well as decreased axonal CaV2.1 abundance and synaptic vesicle exocytosis (Brockhaus et al., 2018). Ectopic Nrxn1α can rescue presynaptic Ca^2+^ influx and synaptic vesicle release, partially through improving CaV2.1 function. Interestingly, Nrxn1α cooperates with α2δ-1, but not α2δ-3, to facilitate presynaptic Ca^2+^ influx in triple *α-Nrxn* KO neurons. When co-expressed in non-neuronal human cells, Nrxn1α drastically increases Ca^2+^ currents through CaV2.1s containing α2δ-1, but not α2δ-3. However, unlike in the previous study (Tong et al., 2017), this study did not find that Nrxn1α could form stable protein complexes with α2δ proteins (Brockhaus et al., 2018). Finally, triple deletion of *α-Nrxns* increases and reduces diffusion coefficients of surface α2δ-1 and α2δ-3, respectively. Altogether, these studies propose models in which α-Nrxns regulate synaptic transmission through modulation of presynaptic Ca^2+^ influx in cooperation with auxiliary α2δ proteins.

Apart from the role of α2δ-2 as a modulator of voltage-gated calcium channels, ectopic presynaptic expression of α2δ-2, but not α2δ-1 or -3, induces mismatched accumulation of postsynaptic inhibitory components including GABA_*A*_Rs apposed to presynaptic excitatory sites (Geisler et al., 2019). Interestingly, this mismatch is drastically potentiated in triple *α-Nrxn* KO cortical neurons, occurring even without ectopic α2δ-2 presynaptic expression (Geisler et al., 2019). It is currently unknown how presynaptic α2δ-2 induces postsynaptic mismatches. Nevertheless, given that α-Nrxns, as well as β-Nrnxs, interact directly with GABA_*A*_R to modulate inhibitory synaptic strength (Zhang et al., 2010), these results suggest the possibility that presynaptic α-Nrxns and α2δ-2 cooperate to regulate postsynaptic recruitment of GABA_*A*_Rs in a trans-synaptic manner (Kang et al., 2008; Geisler et al., 2019).

### 5.5 Dystroglycan (DAG)

The gene *dystroglycan1* (*Dag1*) encodes a single polypeptide that is cleaved into two non-covalently attached proteins: extracellular α-DAG and transmembrane β-DAG (Ibraghimov-Beskrovnaya et al., 1992; Holt et al., 2000). DAG was initially isolated from skeletal muscle as an integral membrane component of the dystrophin-glycoprotein complex (DGC). In humans, defects in α-DAG glycosylation are linked to various progressive muscular dystrophies including Duchenne muscular dystrophy and referred to collectively as α-dystroglycanopathies (Ervasti and Campbell, 1991; Ibraghimov-Beskrovnaya et al., 1992; Cohn and Campbell, 2000; Barresi and Campbell, 2006). Notably, these α-dystroglycanopathies are frequently associated with brain malformation and intellectual disability (Nickolls and Bonnemann, 2018). Structurally, α-DAG contains an N-terminal autonomous folded domain, a central highly O-glycosylated mucin domain, and a globular C-terminal domain (Brancaccio et al., 1995, 1997; Barresi and Campbell, 2006). β-DAG contains a single transmembrane domain and a proline-rich C-terminal cytoplasmic tail (Barresi and Campbell, 2006). In non-neuronal cells, DAG acts by linking extracellular matrix components such as laminin2 to the intracellular actin skeleton through dystrophin (Barresi and Campbell, 2006). In the brain, DAG co-immunoprecipitates with Ig-Nrxn1α fusion proteins and binds the LNS2 and LNS6 domains of α-Nrxns as well as the single LNS domain of β-Nrxns (Sugita et al., 2001; Reissner et al., 2014). Notably, DAG-Nrxn interaction is regulated both by alternative splicing of α-Nrxn and by O-glycosylation of DAG. Insertion at SS2 and SS4 within the LNS2 and LNS6 domains, respectively, of α-Nrxns prevents DAG-Nrxn complex formation, and O-glycosylation of the mucin domain of DAG by LARGE (like-acetyl-glucosaminyl-transferase) is required for binding (Sugita et al., 2001; Reissner et al., 2014). Subsequent biochemical characterization revealed that α-DAG competes with Nxph1 and Nlgn1 for binding to α-Nrxns thereby restricting the formation of α-Nrxn-based multiplexes (Reissner et al., 2014). Conversely, binding of α-DAG to the Nrxn1α LNS2 domain is prevented by Nxph1, although α-DAG and Nxph1 require different epitopes for LNS2 binding (Reissner et al., 2014). The competitive nature of these interactions with α-Nrxn may be a key molecular basis for the formation of distinct α-Nrxn-based trans-synaptic complexes.

Throughout the mouse brain, α-DAG localization seems to be restricted to subsets of GABAergic synapses. In primary rat hippocampal neuron cultures, α-DAG and dystrophin are restricted to GABAergic synapses, and their synaptic localization is established late in development (Levi et al., 2002; Pribiag et al., 2014). In the mouse hippocampal CA1 region, α-DAG is mainly localized in the *stratum pyramidale*, consistent with its role in soma-targeting inhibitory synapses mediated by CCK-expressing INs, but is also found in a subset of GABAergic synapses in the *stratum radiatum* (Fruh et al., 2016; Trotter et al., 2023). In the mouse olfactory bulb (OB), α-DAG is present at large inhibitory synapses in the glomerular layer as well as reciprocal dendrodendritic inhibitory synapses in the external plexiform layer (Trotter et al., 2023). In the cerebellum, α-DAG and β-DAG can be detected in perisomatic and dendritic GABAergic synapses on Purkinje cells, but not on cerebellar INs (Grady et al., 2006; Briatore et al., 2010; Briatore et al., 2020; Trotter et al., 2023). In addition to its localization at inhibitory synapses, DAG is also present in basal lamina and blood vessels, consistent with its function in maintaining blood brain barrier integrity and neuronal and vascular interactions (Tian et al., 1996; Zaccaria et al., 2001; Briatore et al., 2010; Trotter et al., 2023; Tan et al., 2024).

Although DAG is not an α-Nrxn-specific interacting molecule (Sugita et al., 2001), a recent study revealed that the interaction between presynaptic Nrxn3α LNS2 and postsynaptic DAG is required for normal GABAergic transmission in the OB and in the medial prefrontal cortex (mPFC) (Trotter et al., 2023). Deletion of *Nrxn3α/β* in the OB and in the mPFC results in impaired GABAergic transmission by lowering presynaptic release probability, but this could be rescued by expression of just the Nrxn3α LNS2 domain lacking the SS2 insert (LNS2^*SS*2–^). Consistent with the binding studies, inclusion of the SS2 insert abrogated the rescue effect (Sugita et al., 2001; Reissner et al., 2014; Trotter et al., 2023). Furthermore, CRISPR interference or genetic deletion of *Dag1* in OB and mPFC neurons impairs inhibitory, but not excitatory, synaptic transmission by suppressing neurotransmitter release probability, phenocopying the phenotype of *Nrxn3α/β* deletion (Trotter et al., 2023). Interestingly, *Nrxn3* deletion in the mPFC leads to a reduction in mIPSC amplitude that is not rescued by expression of the Nrxn3α LNS2*^SS2–^* construct (Trotter et al., 2023). Furthermore, this decrease is not observed in *Nrxn3* KO OB neurons, nor phenocopied by deletion of *Dag1* in the mPFC, suggesting that regulation of GABA_*A*_R responses by Nrxn3 in the mPFC is DAG-independent (Trotter et al., 2023).

Forebrain-restricted homozygous deletion of *Dag1* from PNs (NEX-Cre and Emx1-Cre mediated deletion) results in drastic defects in formation, maintenance, and transmission of perisomatic inhibitory synapses between CCK-expressing INs and PNs (CCK+ synapses) (Fruh et al., 2016; Jahncke et al., 2024). These defects are specific to CCK+ synapses: loss of *Dag1* has no effect on perisomatic inhibitory synapses formed by PVALB+ INs or excitatory synapses (Fruh et al., 2016; Jahncke et al., 2024). Furthermore, O-linked glycosylation of DAG by protein O-mannosyltransferase 2 (Pomt2), but not the DAG cytoplasmic tail, is required for CCK+ synapse formation (Jahncke et al., 2024), suggesting the involvement of the glycosylated ectodomain of DAG, potentially through Nrxn interaction. In addition, CCK+ INs synaptic terminals are unchanged in *Dag1* T190M KI mice carrying a missense mutation associated with limb-girdle muscular dystrophy (Hara et al., 2011; Fruh et al., 2016) that impairs DAG glycosylation and reduces the ability of recombinant Nrxn proteins to bind to wheat germ agglutinin (WGA)-enriched brain extract (Hara et al., 2011), suggesting that this mutation could impair DAG-Nrxn interaction, although this remains to be tested directly. Furthermore, while two studies reported that the number and localization of CCK+ INs remains unchanged following *Dag1* deletion (Fruh et al., 2016; Jahncke et al., 2024), a third study found that NEX-Cre-mediated *Dag1* deletion leads to a drastic loss of CCK+ INs and their innervation throughout the forebrain (Miller and Wright, 2021). Notably, *Dag1* deletion does not alter the density of PVALB-, somatostatin-, or calretinin-expressing INs (Miller and Wright, 2021). In the cerebellum, conditional deletion of *Dag1* in Purkinje cells (PCs) leads to reduced inhibitory transmission and a decrease in GABAergic synapse formation and maintenance on both the soma and dendrites of PCs (Briatore et al., 2020; Jahncke et al., 2025). These deficits progressively worsen over time, marked by the gradual loss of postsynaptic GABAergic components such as Nlgn2 and GABA_*A*_R, along with increasingly severe inhibitory synapse dysfunction in older mice (Briatore et al., 2020). Consistent with GABAergic synaptic and cerebellar dysfunctions, genetic perturbations of *Dag1* lead to a reduced seizure induction threshold and impaired motor coordination and learning (Grady et al., 2006; Briatore et al., 2020; Jahncke et al., 2024).

In addition to its functions in CCK+ synapses formation and maintenance, DAG also plays a role in inhbitiory synapse plasticity. In cultured hippocampal neurons, α-DAG expression is upregulated by prolonged neuronal activity and is required in a glycosylation-dependent manner for homeostatic upscaling of GABAergic synapses (Pribiag et al., 2014). On the other hand, α-DAG is not required for bicuculine- or tetrodotoxin (TTX)-induced downscaling of glutamatergic and GABAergic synapses, respectively (Pribiag et al., 2014). Treatment with the heparan sulfate proteoglycan (HSPG) agrin, another α-DAG ligand, induces GABAergic synapse upscaling in an α-DAG-dependent manner (Pribiag et al., 2014). Similarly, chronic social defeat stress downregulates α-DAG expression in the ventral hippocampus (vHP) and decreases GABA_*A*_R synaptic transmission, but local administration of agrin into the vHP restores inhibitory synaptic tone and reverses depressive-like behaviors though upregulation of glycosylated α-DAG expression (Xie et al., 2022). Like agrin, Nrxns are also HSPGs (Zhang et al., 2018; Lu et al., 2023), and future studies are needed to address the role of Nrxns in α-DAG-mediated homeostatic scaling of GABAergic synapses.

## 6 α-Neurexins in disease

### 6.1 Neurexins in human diseases: genetic associations and phenotypic outcomes

Mutations in *NRXN* genes, especially *NRXN1*, have been linked to a broad spectrum of psychiatric and neurodevelopmental disorders, most notably schizophrenia (SCZ) and ASD (Bena et al., 2013; Kasem et al., 2018; Hu et al., 2019; Castronovo et al., 2020; Cooper et al., 2024), but also Tourette syndrome (Sundaram et al., 2010; Nag et al., 2013; Huang et al., 2017; Castronovo et al., 2020), intellectual disability (ID) (Castronovo et al., 2020), developmental delay (DD) (Castronovo et al., 2020), substance use disorders (SUD) (Hishimoto et al., 2007; Nussbaum et al., 2008; Stoltenberg et al., 2011), and epilepsy (Perez-Palma et al., 2017; Rochtus et al., 2019). Given the high heritability of SCZ (∼50% concordance in monozygotic twins) (Trifu et al., 2020) and ASD (∼98%) (Grice and Buxbaum, 2006), extensive genetic investigations have been conducted to elucidate their etiology and pathogenesis. Notably, psychiatric disorders are highly heritable, exhibit substantial overlap in their genetic architectures (Brainstorm et al., 2018), and often present co-morbid phenotypes (De Crescenzo et al., 2019).

Initial evidence for the involvement of *NRXN* genes in ASD came from a candidate gene study using single-strand conformation polymorphism (SSCP) analysis, which reported an association between *NRXN1β* and ASD (Feng et al., 2006). In parallel, a study employing array comparative genomic hybridization (array CGH) identified a 250 kb exonic deletion encompassing the promoter and first exon of *NRXN1α* in individuals with SCZ (Kirov et al., 2008). While subsequent research has strengthened the association of *NRXN1–3* with ASD (Reichelt et al., 2012; Vaags et al., 2012; Hu et al., 2019; Khoja et al., 2023), only *NRXN1* has shown a consistent and high-confidence association with SCZ (Reichelt et al., 2012; Kasem et al., 2018; Hu et al., 2019; Tromp et al., 2021). Although one study has suggested a possible link between *NRXN3* singlenucleotide polymorphisms (SNPs) and SCZ (Hu et al., 2013), there is currently no evidence implicating *NRXN2* in SCZ. Importantly, disease-associated genetic variations are largely enriched in the α isoform coding region, rather than in the coding region of the β isoform, across the *NRXN* family (Reichelt et al., 2012; Bena et al., 2013; Hu et al., 2019; Castronovo et al., 2020; Tromp et al., 2021; Cooper et al., 2024).

Exonic deletions in *NRXN1*, particularly those affecting the NRXN1α isoform, have been found to confer substantially elevated risk for schizophrenia, with odds ratios (OR) ranging from 7.44 to 14.4 in large multi-cohort studies (Rujescu et al., 2009; Ikeda et al., 2010; Hu et al., 2019). These deletions are rare but have been identified across multiple populations, including cohorts from Ireland, China, Japan, and the United States. In contrast, common genetic variants in *NRXN1*, such as SNP, do not appear to be widely associated with schizophrenia (Fromer et al., 2014; Purcell et al., 2014; Schizophrenia Working Group of the Psychiatric Genomics, 2014; Hu et al., 2019). However, three studies reported modest but significant associations between *NRXN1* and *NRXN3* polymorphisms and SCZ in a Chinese Han population (Yue et al., 2011; Liu et al., 2012; Hu et al., 2013). Conversely, several studies have reported significant associations between *NRXN1-3* SNPs and ASD in different populations, although further research is required to confirm these findings (Kim et al., 2008; Wang et al., 2009; Yue et al., 2011; Liu et al., 2012; Wang et al., 2018). Therefore, the genetic risk for SCZ, and possibly ASD, conferred by *NRXN1* is attributed primarily to rare, exon-disrupting CNV deletions.

*NRXN1* mutations, particularly monoallelic heterozygous deletions, are characterized by incomplete penetrance and highly variable expressivity. Carriers present with a wide range of phenotypes, including ASD, SCZ, Tourette syndrome, ID, epilepsy, language delay, and mood disorders, underscoring the pleiotropic nature of *NRXN1* (Ching et al., 2010; Schaaf et al., 2012; Bena et al., 2013; Curran et al., 2013; Al Shehhi et al., 2019; Castronovo et al., 2020; Fuccillo and Pak, 2021; Cooper et al., 2024). While some carriers develop neuropsychiatric conditions, others remain asymptomatic (Al Shehhi et al., 2019; Castronovo et al., 2020). These findings suggest that *NRXN1* monoallelic genetic perturbations act more as risk modifiers than deterministic mutations, and their phenotypic outcomes are likely shaped by genetic background and environmental factors. Indeed, genome-wide association studies (GWAS) and recent integrative models suggest that rare variants and polygenic risk may interact to influence neuropsychiatric outcomes (Bergen et al., 2019; Klei et al., 2021). Future studies that combine *NRXN* CNV burden with polygenic risk scores (PRS) will be critical for disentangling the complex genetic architecture of these disorders and for improving risk prediction models.

Bi-allelic *NRXN1* loss-of-function, also referred to as Pitt-Hopkins-like syndrome 2 (OMIM #614325), represents the most severe end of the clinical spectrum associated with *NRXN1* disruption (Zweier et al., 2009; Castronovo et al., 2020; Fuccillo and Pak, 2021). This rare disorder arises from compound inherited heterozygous deletions and/or mutations and is characterized by a consistent phenotype comprising moderate to severe DD or ID, absence of expressive language, severe muscle hypotonia, motor stereotypies, chronic constipation, abnormal sleep-wake cycles, and social interaction deficits (Castronovo et al., 2020). In addition, patients carrying biallelic *NRXN1* loss-of-function mutations often present with breathing abnormalities (Castronovo et al., 2020). To date, only 11 individuals with bi-allelic *NRXN1* disruption have been reported. Most mutations affect the *NRXN1*α isoform and typically span the promoter region and early exons (Castronovo et al., 2020). All but two cases involved variants inherited from asymptomatic parents, underscoring the complexity of genotype–phenotype correlations and the likely contribution of additional genetic or environmental factors (Duong et al., 2012; Castronovo et al., 2020). Altogether, these cases provide compelling evidence that complete loss of NRXN1α function leads to a syndromic neurodevelopmental phenotype with consistent core clinical features and additional variability influenced by environmental and genetic factors.

Another unique case described a female infant with early-onset epileptic encephalopathy and fatal respiratory failure, who carried heterozygous missense mutations in *NRXN1*α (inherited from the mother with a history of sudden infant death syndrome) and *NRXN2*α (from the father with a history of febrile seizures) (Rochtus et al., 2019). Although this digenic combination has only been reported once, the severe respiratory phenotype is consistent with findings from *Nrxn1α/2α* DKO mice, which display impaired central control of breathing, suggesting a potential convergent mechanism of dysfunction (Missler et al., 2003). In addition, postmortem neuropathology also revealed arcuate nucleus hypoplasia and dentate gyrus abnormalities (Rochtus et al., 2019).

### 6.2 Behavioral abnormalities in *α-Nrxn* mutant mice

Studies investigating behavioral deficits in *α-Nrxn* KO rodents have predominantly focused on *Nrxn1α*, with comparatively fewer analyses of *Nrxn2α*, and currently, no behavioral phenotyping data are available for *Nrxn3α* KO rodents (Table 1). Most research employed constitutive deletions of *α-Nrxns*, and factors such as haploinsufficiency and sex-specific effects were inconsistently examined. Additionally, the use of diverse rodent genetic backgrounds may contribute to variability in the observed phenotypes.

**TABLE 1:** Overview of behavioral abnormalities in *α-Nrxns* KO rodents.

Targeted isoforms	Species	Genetic manipulation	Main findings in behavioral abnormalities	References
*Nrxn1α*	Mouse *(Hybrid SV129-C57BL/6)*	Constitutive homozygous KO	• Impaired nest-building behavior • Increased repetitive self-grooming • Prepulse inhibition of startle deficit • Accelerated motor learning • Normal spatial learning and memory • Normal sociability • Normal anxiety	Etherton et al., 2009
*Nrxn1α*	Mouse *(Hybrid SV129-C57BL/6)*	Constitutive heterozygous KO	• Accelerated habituation to novel environments in males • Accelerated habituation to novel objects in males • Normal anxiety	Laarakker et al., 2012
*Nrxn1α*	Mouse *(C57BL/6J)*	Constitutive heterozygous and homozygous KO	• Reduced locomotor activity in homozygous males and females • Increased anxiety in homozygous males • Higher social preference in homozygous males and females • Increased aggressive behaviors in homozygous males • Impaired nest-building behavior in homozygous males and females • Normal spatial learning and memory • Normal short- and long-term working memory • Normal self-grooming • Normal olfaction	Grayton et al., 2013
*Nrxn1α*	Mouse *(C57BL/6J)*	Constitutive homozygous KO	• Reduced fear memory retrieval in males • Normal locomotor activity	Asede et al., 2020
*Nrxn1α*	*Mouse (C57BL/6NCrl)*	Constitutive heterozygous KO	• Impaired social novelty preference • Impaired spatial emotional memory in females • Normal object discrimination memory • Normal locomotor activity • Normal anxiety • Normal prepulse inhibition of startle • Normal nest-building behavior	Dachtler et al., 2015
*Nrxn1α*	Mouse *(C57BL/6N)*	Constitutive homozygous KO and conditional KO (Nex-Cre; telencephalic excitatory neurons)	• Deficits in value-based selection action in constitutive and conditional KO mice • Deficits in value updating and representation of choice value • Disruption of value-associated dorsal striatum neuron activity in conditional KO mice • Normal vision discrimination	Alabi et al., 2020
*Nrxn1α*	Mouse *(Hybrid SV129-C57BL/6)*	Constitutive heterozygous and homozygous KO	• Decreased slow-wave sleep in heterozygous and homozygous KO males (dose-dependent phenotype)	Ostergaard and Kas, 2025
*Nrxn1α*	Mouse *(C57BL/6J)*	Constitutive heterozygous and homozygous KO	• Reduced social novelty preference in heterozygous and homozygous males • Reduced passive interactive behaviors in homozygous females and increased aggressivity in homozygous males • Reduced locomotor activity during dark phase in homozygous males and females • Decreased phase shift upon L/D to D/D change in homozygous males • Increased motor learning and coordination in heterozygous and homozygous males and females • Normal locomotor activity in novel environment • Normal anxiety	Xu et al., 2023
*Nrxn1α*	Mouse *(C57BL/6J****)***	Constitutive heterozygous and homozygous KO	• Reduced isolation-induced USV in homozygous pups • Smaller body weights in homozygous pups and adults • Increased locomotor activity • Delayed developmental milestones • Normal olfaction • Reduced social investigative behaviors in heterozygous and homozygous juvenile and adult males • Increased aggressive behaviors in heterozygous and homozygous adult males • Normal motor learning and coordination • No repetitive behaviors	Armstrong et al., 2020
*Nrxn1α*	Mouse *(C57BL/6J)*	Injection of SCZ patient-derived NRXN1α autoantibodies into the subarachnoid space of the frontal cortex of 8-week-old mice	• Impaired spatial working memory • Prepulse inhibition of startle deficit • Reduced social novelty preference • Impaired novel object discrimination • Normal social preference	Shiwaku et al., 2023
*Nrxn1α*	Rat (Sprague Dawley)	Constitutive homozygous KO	• Increased locomotor activity in males and females • Normal prepulse inhibition of startle but higher startle response • Impaired instrumental conditioning in males • Normal classical conditioning • Impaired latent inhibition • Impaired spatial learning in males and females	Esclassan et al., 2015
*Nrxn1α*	Rat (Sprague Dawley)	Constitutive homozygous KO	• Increased locomotor activity • Increased gamma power and gamma coherence in cortico-striatal and thalamocortical circuits (freely moving animals) • Reduced auditory-evoked theta oscillation in frontal and parietal cortical regions • Profound defects in auditory mismatch negativity responses • Normal sociability and social stimulus-driven neuronal oscillations	Janz et al., 2022
*Nrxn1α*	Rat (Sprague Dawley)	Constitutive homozygous KO	• Delayed auditory brainstem responses in juvenile but not adult rats • Normal hearing sensitivities	Marashli et al., 2024
*Nrxn1α*	Rat (Sprague Dawley)	Constitutive homozygous KO	• Increased locomotor activity • Increased social play behavior • Increased age-inappropriate sexual mounting	Achterberg et al., 2025
*Nrxn1α*	Rat (Sprague Dawley)	Constitutive heterozygous and homozygous KO	• Reduced isolation-induced USV in homozygous male and female pups • Increased locomotor activity in novel environment in homozygous juvenile males • Reduced social play behaviors in homozygous juvenile males • Reduced prosocial helping behaviors in heterozygous and homozygous juvenile males and females • Reduced performance in food-reward task in heterozygous and homozygous juvenile males and females • Facilitated nurturing behaviors toward isolated pups • Increased object investigation in homozygous males • Normal olfaction • Normal social preference and social novelty preference	Kight et al., 2021
*Nrxn2α*	Mouse *(C57BL/6NCrl)*	Constitutive homozygous KO	• Impaired sociability and social novel preference • Normal locomotor activity • Heightened anxiety • Normal prepulse inhibition of startle • Normal spatial emotional memory • No depression-related behaviors • Normal olfaction	Dachtler et al., 2014
*Nrxn2α*	Mouse *(C57BL/6NCrl)*	Constitutive heterozygous KO	• Impaired social novelty preference • Impaired object discrimination memory • Normal spatial emotional memory • Normal locomotor activity • Normal anxiety • Normal prepulse inhibition of startle • Normal nest-building behavior	Dachtler et al., 2015
*Nrxn2α*	*Mouse (C57BL/6J)*	Constitutive heterozygous and homozygous KO	• Heightened anxiety in homozygous males and females • Increased repetitive self-grooming in homozygous females • Impaired nest-building behavior • Normal locomotor activity • Impaired sociability and social novel preference in homozygous females • Normal spatial learning and memory • Normal olfaction	Born et al., 2015
Nrxn3α	Rat (Long Evans)	shRNA-mediated knockdown of Nrxn3α in the central amygdala	• Increased Varicella-zoester virus-associated pain response in male and proestrus female rats	Kramer et al., 2022; Kramer et al., 2024
*Nrxn1α*, *Nrxn2α*, *Nrxn3α*	Mouse *(Hybrid SV129-C57BL/6)*	• Constitutive homozygous *Nrxn1α/2α* and *Nrxn2α/3α* DKO	• Faster saturation of maximum oxygen uptake during physical exercise • Normal maximum oxygen uptake during physical exercise • Normal ventilation frequency • Normal auditory threshold	Sons et al., 2006

Commonly reported behavioral abnormalities in *α-Nrxn* KO mice include increased anxiety-like behavior, impaired nest-building, and elevated self-grooming (Etherton et al., 2009; Grayton et al., 2013; Dachtler et al., 2014; Born et al., 2015; Dachtler et al., 2015). However, it remains unclear whether the increased self-grooming reflects motor stereotypies or anxiety-induced behavior (Liu H. et al., 2021). These phenotypes are reminiscent of traits observed in individuals with ASD and SCZ (Silverman et al., 2010; Angoa-Perez et al., 2013; Kazdoba et al., 2016; Simmons et al., 2021). Notably, findings regarding social deficits in *α-Nrxn* KO mice are inconsistent: while some studies report no change in sociability (Etherton et al., 2009; Janz et al., 2022), others describe alterations in specific social behaviors (Grayton et al., 2013; Dachtler et al., 2014, 2015; Born et al., 2015; Achterberg et al., 2025).

Atypical sensory processing is frequently observed in patients with neurodevelopmental and psychiatric disorders (Piek and Dyck, 2004; Javitt, 2009; Marco et al., 2011; Gigliotti et al., 2024) as well as in corresponding animal models (Braff and Geyer, 1990; Balasco et al., 2019; Falcao et al., 2024). Although one study reported deficits in prepulse inhibition (PPI) of the startle response in homozygous *Nrxn1α* KO mice (Etherton et al., 2009), several other studies found normal PPI in both *Nrxn1α* and *Nrxn2α* KO rodents (Dachtler et al., 2014, 2015; Esclassan et al., 2015). Interestingly, *Nrxn1α* KO adult rats display normal PPI but exhibit an increased baseline startle response (Esclassan et al., 2015), along with reduced auditory-evoked theta oscillation in frontal and parietal cortical regions and profound defects in auditory mismatch negativity responses (Janz et al., 2022). In addition, altered auditory brainstem responses have been observed in juvenile, but not adult, *Nrxn1α* KO rats. Collectively, these findings suggest that deletion of *Nrxn1α* or *Nrxn2α* does not impair sensorimotor gating but does affect auditory stimulus processing. Moreover, olfactory function appears to be preserved in *Nrxn1α* KO mice and rats (Laarakker et al., 2012; Grayton et al., 2013; Kight et al., 2021), as well as in *Nrxn2α* KO mice (Dachtler et al., 2014; Born et al., 2015). However, whether loss of *α-Nrxns* affects other sensory modalities, such as somatosensory or visual processing, remains unclear. Given that these functions are frequently affected in individuals with ASD (Wang et al., 2015; Shafer et al., 2021), and in related mouse models (Chelini et al., 2019; Cheng et al., 2020), further investigation is warranted to determine the broader impact of α-Nrxn deletion on sensory system function.

Despite notable functional synaptic deficits in the cortex and hippocampus (Missler et al., 2003; Kattenstroth et al., 2004; Uchigashima et al., 2020), *Nrxn1α* KO mice and *Nrxn2α* KO mice typically exhibit modest cognitive impairments (Kattenstroth et al., 2004; Dudanova et al., 2007; Etherton et al., 2009; Dachtler et al., 2014; Aoto et al., 2015; Born et al., 2015; Uchigashima et al., 2020; Trotter et al., 2023). Specifically, *Nrxn1α* KO mice display normal spatial and working memory in Morris water maze and novel object recognition tests (Etherton et al., 2009; Grayton et al., 2013; Dachtler et al., 2015), although they show fear memory deficits in both classical and instrumental conditioning paradigms (Dachtler et al., 2015; Asede et al., 2020). On the other hand, deletion of *Nrxn2α* in mice does not affect fear memory (Dachtler et al., 2014, 2015). These studies suggest that *Nrxn1α*, but not *Nrxn2α*, may selectively regulate fear-related learning and memory. In contrast, *Nrxn1α* KO rats show impairments in instrumental, but not classical, conditioning, and exhibit deficits in latent inhibition and spatial memory, indicating species-dependent behavioral outcomes, even among rodents (Esclassan et al., 2015).

### 6.3 Modeling α-NRXN-linked neurodevelopmental disorders using hiPSC-derived cellular systems

Although animal models have significantly advanced our understanding of the roles of Nrxns in the nervous system, they face several key limitations in faithfully recapitulating certain aspects of neurodevelopmental and psychiatric disorders associated with NRXN perturbations. Indeed, despite the absence of electrophysiological deficits after heterozygous *Nrxn1* deletion in mouse neurons engineered from ES cells, iPSC-derived human *NRXN1*^+/–^ neurons exhibit robust alterations in excitatory neurotransmission, suggesting that synaptic functions of Nrxns may be species-dependent (Pak et al., 2015, 2021). Furthermore, the penetrance of heterozygous *NRXN* deletions in humans is incomplete, and the associated clinical presentations are highly variable (Castronovo et al., 2020; Fuccillo and Pak, 2021; Cooper et al., 2024), suggesting that additional genetic co-factors likely contribute to the resulting phenotype. Dysregulation of alternative splicing is thought to be an essential molecular mechanism for the pathogenesis of several neurodevelopmental and psychiatric disorders (Zhang et al., 2022; Ushkaryov et al., 1994; Dando et al., 2024), and NRXNs may exhibit species-specific alternative splicing that could be further regulated by patient genetic determinants. Indeed, analysis of basal and activity-dependent exon splicing in human and mouse neurons revealed significant differences between the two species (Dando et al., 2024). In addition, hiPSC-derived forebrain neurons and astroglia exhibit a diverse repertoire of NRXN1α isoforms, reflecting the extensive alternative splicing of NRXN1α observed in the human brain (Flaherty et al., 2019). Consequently, for elucidating the role of NRXNs in diverse human neurodevelopmental and psychiatric conditions, it is essential to use patient-derived cellular models, including human iPSC-derived neurons and other relevant brain cell types.

An unexpected finding from hiPSC studies is that exonic deletion of *NRXN1*α skews fate choice in neural progenitors and perturbs neuronal and glial maturation and the functions of these cells (Flaherty et al., 2019; Lam et al., 2019; Bose et al., 2025). Interestingly, NRXN1α expression is upregulated during neural induction and neuronal differentiation (Lam et al., 2019) suggesting it plays a pivotal role in establishment of neural stem cells, in neuronal differentiation, and in maturation of functional neuronal and glial cells. Both bi-allelic and heterozygous *NRXN1*α deletion in hiPSC-derived neurons and brain organoids alter neuronal and glial faith and differentiation, impair neuronal maturation, and hinder the emergence of mature excitatory neurons (Flaherty et al., 2019; Lam et al., 2019; Sebastian et al., 2023; Bose et al., 2025). On the other hand, a previous study has reported that knockdown of NRXN1α in hiPSCs selectively affects astrocytic, but not neuronal, differentiation and maturation (Zeng et al., 2013). Bi-allelic *NRXN1*α deletion in microglia impairs their ability to support neuronal differentiation, maturation, and the development of neuronal networks in differentiating iPSC-derived neuroepithelial stem (NES) cells (Bose et al., 2025). This impairment is driven by increased secretion of interleukin-6 (IL-6) from *NRXN1*α-deficient microglia, which negatively affects neuronal maturation and function (Bose et al., 2025). Interestingly, human brain organoids with engineered heterozygous *NRXN1*α deletion followed similar developmental trajectories as controls, with only subtle differences emerging at 3.5 months and in both glial and neuronal populations (Sebastian et al., 2023). In contrast, organoids derived from SCZ donors carrying heterozygous *NRXN1*α deletions showed profound developmental perturbations as early as 3 weeks, which persisted throughout maturation (Sebastian et al., 2023). These disruptions impacted not only glial and neuronal populations but also neural progenitor cells (Sebastian et al., 2023). Although both engineered and donor-derived *NRXN1*α deletions altered gene networks associated with the unfolded protein response (UPR) and RNA splicing, these changes were more pronounced in samples with donor-derived deletions (Sebastian et al., 2023). Overall, while some changes in gene expression and developmental abnormalities are shared in isogenic and SCZ *NRXN1*α deletion contexts, they emerge at different developmental stages and affect distinct cell types. Altogether, these findings support a role for α-NRXNs, in regulating neuronal and microglial differentiation and maturation. Across multiple experimental systems, loss or reduction of NRXN1α disrupts neural lineage specification, impairs neuronal maturation and alters glial cell fate. These converging findings support that α-NRXNs are essential regulators of neurodevelopmental processes in hiPSC-derived systems.

Consistent with the canonical role of NRXN1α in synaptic transmission and its involvement in neuronal maturation, genetic perturbations of *NRXN1*α in patient iPSC-derived neurons impact neuronal activity. While most of the studies report reduced neuronal activity, impaired network synchrony, and diminished Ca^2+^ signaling (Flaherty et al., 2019; Lam et al., 2019; Sebastian et al., 2023; Bose et al., 2025; Fernando et al., 2025), some have observed increased excitability, elevated sodium currents (Avazzadeh et al., 2021), and enhanced Ca^2+^ transients (Avazzadeh et al., 2019). This increased activity is associated with the upregulation of gene networks involved in ion transport, including VGCCs (Avazzadeh et al., 2019, 2021).

Recent studies have demonstrated that the pathogenic effects of *NRXN1* deletions are highly dependent on their genomic position. Using hiPSCs derived from individuals diagnosed with psychotic disorders and carrying rare heterozygous intragenic deletions in either the 5′ region (exons 1–2) or 3′ region (exons 21–23) of *NRXN1*, researchers uncovered distinct molecular mechanisms underlying impaired neuronal activity (Flaherty et al., 2019; Fernando et al., 2025). Deletions at the 5′ end led to a significant reduction in canonical NRXN1α isoforms, consistent with a haploinsufficiency model (Flaherty et al., 2019; Fernando et al., 2025). In contrast, 3′ deletions resulted in the production of multiple aberrant NRXN1α splice isoforms that were absent in control hiPSC-derived neurons and post-mortem human brain tissue, implicating a GOF mechanism (Flaherty et al., 2019; Fernando et al., 2025). Despite these differences, both 5′ and 3′ deletions caused reduced spontaneous excitatory activity, impaired synaptic transmission, and disrupted neuronal maturation (Flaherty et al., 2019; Fernando et al., 2025). Importantly, overexpression of wild-type NRXN1α or treatment with β-estradiol (to increase *NRXN1*α locus expression) rescued neuronal deficits in neurons with 5′ deletions, supporting a LOF etiology (Flaherty et al., 2019; Fernando et al., 2025). However, the same approach failed to rescue phenotypes in neurons with 3′ deletions (Flaherty et al., 2019). Moreover, overexpression of mutant NRXN1α isoforms in control hiPSC-neurons suppressed neuronal activity, suggesting that these aberrant splice variants exert dominant-negative effects (Flaherty et al., 2019). Finally, antisense oligonucleotides targeting 3’ deletion mutant isoforms to significantly reduce the abundance of mutant isoforms led to robust changes in genes expression enriched for synaptic properties, neurotransmitter signaling and neurodevelopmental pathways (Fernando et al., 2025). Together, these findings underscore that both haploinsufficiency of wild-type *NRXN1*α and dominant effects of mutant isoforms contribute to the functional and clinical heterogeneity observed in *NRXN1*-related disorders, emphasizing the importance of isoform-level resolution in mechanistic studies and therapeutic design.

Together, hiPSC-based cellular models are powerful tools for probing the causal effects of *α-NRXN* genetic perturbations in neurodevelopmental and psychiatric disorders, as they allow precise experimental manipulation and, in some cases, rescue of disease-associated phenotypes. However, these systems have notable limitations, including inherent variability between lines, the lack of physiological microenvironments with multiple interacting cell types, and the absence of vasculature, which can create abnormal metabolic states. Crucially, hiPSC models cannot capture behavioral phenotypes, a hallmark of many psychiatric and neurological disorders. Hence, while hiPSCs are promising models for studying neurodevelopmental and psychiatric disorders, including *α-NRXN*–related perturbations, humanized rodent and non-human primate models remain essential complementary approaches.

## 7 Concluding remarks

Despite decades of investigation, the precise roles of α-Nrxns in neural and synaptic function and disease remain incompletely understood. These large, polymorphic synaptic adhesion molecules are distinguished from their shorter β-counterparts by extensive extracellular regions containing multiple additional LNS domains and EGF domains. These LNS domains are thought to further mediate additional context-dependent ligand interactions. Although both types of isoform share the membrane-proximal LNS domain, trans-membrane region, and identical intracellular tails, suggesting some level of redundancy in core functions, α-Nrxns appear uniquely capable of regulating presynaptic calcium channel activity and neurotransmitter release, indicating specialized roles in synaptic transmission.

Yet, functional distinctions between α- and β-Nrxns remain difficult to disentangle, as observed differences may reflect divergent spatiotemporal expression patterns rather than intrinsic molecular properties. The generation and characterization of isoform-specific tools such as selective conditional knockouts or epitope-tagged knock-in models are important as future studies to further define α-Nrxn-specific contributions *in vivo*.

α-Nrxns exhibit highly context-dependent roles in synaptic function, with their effects varying according to cell type, synapse type and brain region. For example, loss of *Nrxn1α* selectively impairs NMDAR-mediated transmission at thalamostriatal synapses, while corticostriatal and hippocampal synapses remain unaffected (Etherton et al., 2009; Davatolhagh and Fuccillo, 2021). Similarly, triple *α-Nrxn* deletion profoundly reduces GABAergic transmission in brainstem and neocortical synapses (Missler et al., 2003; Zhang et al., 2005), whereas deletion of individual *α-Nrxn* isoforms produces modest or highly specific effects, such as the Nrxn1α–Nlgn3 complex selectively regulating inhibition from CCK-expressing INs onto hippocampal PNs (Uchigashima et al., 2020). Such context-dependent roles likely arise from a combination of factors, including the local expression of specific α-Nrxn binding partners, differential expression of alternative α-Nrxn splice isoforms, or even intrinsic sexual dimorphism at particular synapses, collectively enabling α-Nrxns to fine-tune synaptic transmission in a cell- and synapse-specific manner.

Emerging evidence suggests that *α-NRXNs* play a more prominent role than β-isoforms in the pathogenesis of neurodevelopmental and neuropsychiatric disorders, including ASD and SCZ, where synaptic dysfunction is a central pathology. Notably, recent studies using iPSC-derived human neurons have suggested that genetic perturbations of *α-NRXNs* may interact with patient-specific genetic backgrounds and impair neuronal maturation prior to synapse formation. Furthermore, distinct *α-NRXN* deletions contribute to disease through diverse mechanisms, underscoring the need to stratify patients by whether their mutations act via LOF or GOF effects. Such stratification is critical to developing individualized strategies aimed at restoring NRXN dysfunction and dysregulation by either increasing WT isoform expression or suppressing pathogenic variants.

To further our understanding of the physiological and pathological relevance of α-*NRXNs*, future work must combine isoform-specific genetic models, high-resolution expression profiling, and context-dependent functional assays across diverse neural systems. Only through such integrative approaches can we define the essential, redundant, and disease-relevant roles of *α-NRXNS*, ultimately informing targeted therapeutic strategies.

## References

[B1] Achterberg E. J. M. Biemans B. Vanderschuren L. (2025). Neurexin1alpha knockout in rats causes aberrant social behaviour: Relevance for autism and schizophrenia. *Psychopharmacology* 242 1069–1089. 10.1007/s00213-024-06559-z 38418646 PMC12043747

[B2] Akopian G. Walsh J. P. (2002). Corticostriatal paired-pulse potentiation produced by voltage-dependent activation of NMDA receptors and L-type Ca(2+) channels. *J. Neurophysiol.* 87 157–165. 10.1152/jn.00115.2001 11784738

[B3] Al Shehhi M. Forman E. B. Fitzgerald J. E. McInerney V. Krawczyk J. Shen S. (2019). NRXN1 deletion syndrome; phenotypic and penetrance data from 34 families. *Eur. J. Med. Genet.* 62 204–209. 10.1016/j.ejmg.2018.07.015 30031152

[B4] Alabi O. O. Davatolhagh M. F. Robinson M. Fortunato M. P. Vargas Cifuentes L. Kable J. W. (2020). Disruption of Nrxn1alpha within excitatory forebrain circuits drives value-based dysfunction. *Elife* 9:e54838. 10.7554/eLife.54838 33274715 PMC7759380

[B5] Andersen S. L. (2003). Trajectories of brain development: Point of vulnerability or window of opportunity? *Neurosci. Biobehav. Rev.* 27 3–18. 10.1016/s0149-7634(03)00005-8 12732219

[B6] Anderson G. R. Aoto J. Tabuchi K. Foldy C. Covy J. Yee A. X. (2015). beta-neurexins control neural circuits by regulating synaptic endocannabinoid signaling. *Cell* 162 593–606. 10.1016/j.cell.2015.06.056 26213384 PMC4709013

[B7] Angoa-Perez M. Kane M. J. Briggs D. I. Francescutti D. M. Kuhn D. M. (2013). Marble burying and nestlet shredding as tests of repetitive, compulsive-like behaviors in mice. *J. Vis. Exp.* 82:50978. 10.3791/50978 24429507 PMC4108161

[B8] Aoto J. Foldy C. Ilcus S. M. Tabuchi K. Sudhof T. C. (2015). Distinct circuit-dependent functions of presynaptic neurexin-3 at GABAergic and glutamatergic synapses. *Nat. Neurosci.* 18 997–1007. 10.1038/nn.4037 26030848 PMC4482778

[B9] Aoto J. Martinelli D. C. Malenka R. C. Tabuchi K. Sudhof T. C. (2013). Presynaptic neurexin-3 alternative splicing trans-synaptically controls postsynaptic AMPA receptor trafficking. *Cell* 154 75–88. 10.1016/j.cell.2013.05.060 23827676 PMC3756801

[B10] Armstrong E. C. Caruso A. Servadio M. Andreae L. C. Trezza V. Scattoni M. L. (2020). Assessing the developmental trajectory of mouse models of neurodevelopmental disorders: Social and communication deficits in mice with Neurexin 1alpha deletion. *Genes Brain Behav.* 19:e12630. 10.1111/gbb.12630 31823470

[B11] Asede D. Joseph A. Bolton M. M. (2020). Deletion of NRXN1alpha impairs long-range and local connectivity in amygdala fear circuit. *Transl. Psychiatry* 10:242. 10.1038/s41398-020-00926-y 32684634 PMC7370229

[B12] Avazzadeh S. McDonagh K. Reilly J. Wang Y. Boomkamp S. D. McInerney V. (2019). Increased Ca(2+) signaling in NRXN1alpha(+/-) neurons derived from ASD induced pluripotent stem cells. *Mol. Autism.* 10:52. 10.1186/s13229-019-0303-3 31893021 PMC6937972

[B13] Avazzadeh S. Quinlan L. R. Reilly J. McDonagh K. Jalali A. Wang Y. (2021). NRXN1alpha(+/-) is associated with increased excitability in ASD iPSC-derived neurons. *BMC Neurosci.* 22:56. 10.1186/s12868-021-00661-0 34525970 PMC8442436

[B14] Balasco L. Provenzano G. Bozzi Y. (2019). Sensory abnormalities in autism spectrum disorders: A focus on the tactile domain, from genetic mouse models to the clinic. *Front. Psychiatry* 10:1016. 10.3389/fpsyt.2019.01016 32047448 PMC6997554

[B15] Bampton E. T. Ma C. H. Tolkovsky A. M. Taylor J. S. (2005). Osteonectin is a Schwann cell-secreted factor that promotes retinal ganglion cell survival and process outgrowth. *Eur. J. Neurosci.* 21 2611–2623. 10.1111/j.1460-9568.2005.04128.x 15926910

[B16] Barresi R. Campbell K. P. (2006). Dystroglycan: from biosynthesis to pathogenesis of human disease. *J. Cell. Sci.* 119 199–207. 10.1242/jcs.02814 16410545

[B17] Beglopoulos V. Montag-Sallaz M. Rohlmann A. Piechotta K. Ahmad M. Montag D. (2005). Neurexophilin 3 is highly localized in cortical and cerebellar regions and is functionally important for sensorimotor gating and motor coordination. *Mol. Cell. Biol.* 25 7278–7288. 10.1128/MCB.25.16.7278-7288.2005 16055736 PMC1190246

[B18] Bena F. Bruno D. L. Eriksson M. van Ravenswaaij-Arts C. Stark Z. Dijkhuizen T. (2013). Molecular and clinical characterization of 25 individuals with exonic deletions of NRXN1 and comprehensive review of the literature. *Am. J. Med. Genet. B Neuropsychiatr. Genet.* 162B 388–403. 10.1002/ajmg.b.32148 23533028

[B19] Bergen S. E. Ploner A. Howrigan D. O’Donovan M. C. (2019). Joint contributions of rare copy number variants and common SNPs to risk for schizophrenia. *Am. J. Psychiatry* 176 29–35. 10.1176/appi.ajp.2018.17040467 30392412 PMC6408268

[B20] Berninghausen O. Rahman M. A. Silva J. P. Davletov B. Hopkins C. Ushkaryov Y. A. (2007). Neurexin Ibeta and neuroligin are localized on opposite membranes in mature central synapses. *J. Neurochem.* 103 1855–1863. 10.1111/j.1471-4159.2007.04918.x 17868325 PMC2517655

[B21] Bernstein G. M. Jones O. T. (2007). Kinetics of internalization and degradation of N-type voltage-gated calcium channels: Role of the alpha2/delta subunit. *Cell. Calcium* 41 27–40. 10.1016/j.ceca.2006.04.010 16759698

[B22] Born G. Breuer D. Wang S. Rohlmann A. Coulon P. Vakili P. (2014). Modulation of synaptic function through the alpha-neurexin-specific ligand neurexophilin-1. *Proc. Natl. Acad. Sci. U S A.* 111 E1274–E1283. 10.1073/pnas.1312112111 24639499 PMC3977266

[B23] Born G. Grayton H. M. Langhorst H. Dudanova I. Rohlmann A. Woodward B. W. (2015). Genetic targeting of NRXN2 in mice unveils role in excitatory cortical synapse function and social behaviors. *Front. Synaptic Neurosci.* 7:3. 10.3389/fnsyn.2015.00003 25745399 PMC4333794

[B24] Bose R. Posada-Perez M. Karvela E. Skandik M. Keane L. Falk A. (2025). Bi-allelic NRXN1alpha deletion in microglia derived from iPSC of an autistic patient increases interleukin-6 production and impairs supporting function on neuronal networking. *Brain Behav. Immun.* 123 28–42. 10.1016/j.bbi.2024.09.001 39243986

[B25] Boucard A. A. Chubykin A. A. Comoletti D. Taylor P. Sudhof T. C. (2005). A splice code for trans-synaptic cell adhesion mediated by binding of neuroligin 1 to alpha- and beta-neurexins. *Neuron* 48 229–236. 10.1016/j.neuron.2005.08.026 16242404

[B26] Boxer E. E. Seng C. Lukacsovich D. Kim J. Schwartz S. Kennedy M. J. (2021). Neurexin-3 defines synapse- and sex-dependent diversity of GABAergic inhibition in ventral subiculum. *Cell. Rep.* 37:110098. 10.1016/j.celrep.2021.110098 34879268 PMC8763380

[B27] Bradshaw A. D. (2012). Diverse biological functions of the SPARC family of proteins. *Int. J. Biochem. Cell. Biol.* 44 480–488. 10.1016/j.biocel.2011.12.021 22249026 PMC3312742

[B28] Braff D. L. Geyer M. A. (1990). Sensorimotor gating and schizophrenia. Human and animal model studies. *Arch. Gen. Psychiatry* 47 181–188. 10.1001/archpsyc.1990.01810140081011 2405807

[B29] Brainstorm C. Anttila V. Bulik-Sullivan B. Finucane H. K. Walters R. K. Bras J. (2018). Analysis of shared heritability in common disorders of the brain. *Science* 360:6395. 10.1126/science.aap8757 29930110 PMC6097237

[B30] Brancaccio A. Schulthess T. Gesemann M. Engel J. (1995). Electron microscopic evidence for a mucin-like region in chick muscle alpha-dystroglycan. *FEBS Lett.* 368 139–142. 10.1016/0014-5793(95)00628-m 7615068

[B31] Brancaccio A. Schulthess T. Gesemann M. Engel J. (1997). The N-terminal region of alpha-dystroglycan is an autonomous globular domain. *Eur. J. Biochem.* 246 166–172. 10.1111/j.1432-1033.1997.00166.x 9210479

[B32] Brayman V. L. Taetzsch T. Miko M. Dahal S. Risher W. C. Valdez G. (2021). Roles of the synaptic molecules Hevin and SPARC in mouse neuromuscular junction development and repair. *Neurosci. Lett.* 746:135663. 10.1016/j.neulet.2021.135663 33493647 PMC8418192

[B33] Briatore F. Patrizi A. Viltono L. Sassoe-Pognetto M. Wulff P. (2010). Quantitative organization of GABAergic synapses in the molecular layer of the mouse cerebellar cortex. *PLoS One* 5:e12119. 10.1371/journal.pone.0012119 20711348 PMC2920831

[B34] Briatore F. Pregno G. Di Angelantonio S. Frola E. De Stefano M. E. Vaillend C. (2020). Dystroglycan mediates clustering of essential GABAergic components in cerebellar purkinje cells. *Front. Mol. Neurosci.* 13:164. 10.3389/fnmol.2020.00164 32982691 PMC7485281

[B35] Brockhaus J. Kahl I. Ahmad M. Repetto D. Reissner C. Missler M. (2024). Conditional knockout of neurexins alters the contribution of calcium channel subtypes to presynaptic Ca(2+) influx. *Cells* 13:981. 10.3390/cells13110981 38891114 PMC11171642

[B36] Brockhaus J. Schreitmuller M. Repetto D. Klatt O. Reissner C. Elmslie K. (2018). alpha-neurexins together with alpha2delta-1 auxiliary subunits regulate Ca(2+) influx through Ca(v)2.1 channels. *J. Neurosci.* 38 8277–8294. 10.1523/JNEUROSCI.0511-18.2018 30104341 PMC6596161

[B37] Brown S. P. Safo P. K. Regehr W. G. (2004). Endocannabinoids inhibit transmission at granule cell to Purkinje cell synapses by modulating three types of presynaptic calcium channels. *J. Neurosci.* 24 5623–5631. 10.1523/JNEUROSCI.0918-04.2004 15201335 PMC6729326

[B38] Cabral-Miranda F. Araujo A. P. B. Medinas D. B. Gomes F. C. A. (2025). Astrocytic Hevin/SPARCL-1 regulates cognitive decline in pathological and normal brain aging. *Aging Cell* 24:e14493. 10.1111/acel.14493 39935382 PMC12074016

[B39] Cahoy J. D. Emery B. Kaushal A. Foo L. C. Zamanian J. L. Christopherson K. S. (2008). A transcriptome database for astrocytes, neurons, and oligodendrocytes: A new resource for understanding brain development and function. *J. Neurosci.* 28 264–278. 10.1523/JNEUROSCI.4178-07.2008 18171944 PMC6671143

[B40] Canti C. Nieto-Rostro M. Foucault I. Heblich F. Wratten J. Richards M. W. (2005). The metal-ion-dependent adhesion site in the Von Willebrand factor-A domain of alpha2delta subunits is key to trafficking voltage-gated Ca2+ channels. *Proc. Natl. Acad. Sci. U S A.* 102 11230–11235. 10.1073/pnas.0504183102 16061813 PMC1183569

[B41] Castronovo P. Baccarin M. Ricciardello A. Picinelli C. Tomaiuolo P. Cucinotta F. (2020). Phenotypic spectrum of NRXN1 mono- and bi-allelic deficiency: A systematic review. *Clin. Genet.* 97 125–137. 10.1111/cge.13537 30873608

[B42] Chelini G. Zerbi V. Cimino L. Grigoli A. Markicevic M. Libera F. (2019). Aberrant somatosensory processing and connectivity in mice lacking engrailed-2. *J. Neurosci.* 39 1525–1538. 10.1523/JNEUROSCI.0612-18.2018 30593497 PMC6381254

[B43] Chen F. Venugopal V. Murray B. Rudenko G. (2011). The structure of neurexin 1alpha reveals features promoting a role as synaptic organizer. *Structure* 19 779–789. 10.1016/j.str.2011.03.012 21620716 PMC3134934

[B44] Chen G. Xu J. Luo H. Luo X. Singh S. K. Ramirez J. J. (2022). Hevin/Sparcl1 drives pathological pain through spinal cord astrocyte and NMDA receptor signaling. *JCI Insight* 7:161028. 10.1172/jci.insight.161028 36256481 PMC9746899

[B45] Chen J. Li L. Chen S. R. Chen H. Xie J. D. Sirrieh R. E. (2018). The alpha2delta-1-NMDA receptor complex is critically involved in neuropathic pain development and gabapentin therapeutic actions. *Cell. Rep.* 22 2307–2321. 10.1016/j.celrep.2018.02.021 29490268 PMC5873963

[B46] Cheng N. Pagtalunan E. Abushaibah A. Naidu J. Stell W. K. Rho J. M. (2020). Atypical visual processing in a mouse model of autism. *Sci. Rep.* 10:12390. 10.1038/s41598-020-68589-9 32709898 PMC7381655

[B47] Cheng S. Seven A. B. Wang J. Skiniotis G. Ozkan E. (2016). Conformational plasticity in the transsynaptic neurexin-cerebellin-glutamate receptor adhesion complex. *Structure* 24 2163–2173. 10.1016/j.str.2016.11.004 27926833 PMC5149402

[B48] Ching M. S. Shen Y. Tan W. H. Jeste S. S. Morrow E. M. Chen X. (2010). Deletions of NRXN1 (neurexin-1) predispose to a wide spectrum of developmental disorders. *Am. J. Med. Genet. B Neuropsychiatr. Genet.* 153B 937–947. 10.1002/ajmg.b.31063 20468056 PMC3001124

[B49] Chofflet N. Naito Y. Pastore A. J. Padmanabhan N. Nguyen P. T. Poitras C. (2024). Structural and functional characterization of the IgSF21-neurexin2alpha complex and its related signaling pathways in the regulation of inhibitory synapse organization. *Front. Mol. Neurosci.* 17:1371145. 10.3389/fnmol.2024.1371145 38571813 PMC10989685

[B50] Cohn R. D. Campbell K. P. (2000). Molecular basis of muscular dystrophies. *Muscle Nerve* 23 1456–1471. 10.1002/1097-4598(200010)23:10<1456::aid-mus2<3.0.co;2-t 11003781

[B51] Comoletti D. Miller M. T. Jeffries C. M. Wilson J. Demeler B. Taylor P. (2010). The macromolecular architecture of extracellular domain of alphaNRXN1: domain organization, flexibility, and insights into trans-synaptic disposition. *Structure* 18 1044–1053. 10.1016/j.str.2010.06.005 20696403 PMC2948785

[B52] Cooper J. N. Mittal J. Sangadi A. Klassen D. L. King A. M. Zalta M. (2024). Landscape of NRXN1 gene variants in phenotypic manifestations of autism spectrum disorder: A systematic review. *J. Clin. Med.* 13:2067. 10.3390/jcm13072067 38610832 PMC11012327

[B53] Curran S. Ahn J. W. Grayton H. Collier D. A. Ogilvie C. M. (2013). NRXN1 deletions identified by array comparative genome hybridisation in a clinical case series - further understanding of the relevance of NRXN1 to neurodevelopmental disorders. *J. Mol. Psychiatry* 1:4. 10.1186/2049-9256-1-4 25408897 PMC4223877

[B54] Dachtler J. Glasper J. Cohen R. N. Ivorra J. L. Swiffen D. J. Jackson A. J. (2014). Deletion of alpha-neurexin II results in autism-related behaviors in mice. *Transl. Psychiatry* 4:e484. 10.1038/tp.2014.123 25423136 PMC4259993

[B55] Dachtler J. Ivorra J. L. Rowland T. E. Lever C. Rodgers R. J. Clapcote S. J. (2015). Heterozygous deletion of alpha-neurexin I or alpha-neurexin II results in behaviors relevant to autism and schizophrenia. *Behav. Neurosci.* 129 765–776. 10.1037/bne0000108 26595880 PMC4655861

[B56] Dai J. Aoto J. Sudhof T. C. (2019). Alternative splicing of presynaptic neurexins differentially controls postsynaptic NMDA and AMPA receptor responses. *Neuron* 102 993–1008.e1005. 10.1016/j.neuron.2019.03.032 31005376 PMC6554035

[B57] Dai J. Liakath-Ali K. Golf S. R. Sudhof T. C. (2023). Correction: Distinct neurexin-cerebellin complexes control AMPA- and NMDA-receptor responses in a circuit-dependent manner. *Elife* 12:e94305. 10.7554/eLife.94305 37943030 PMC10635641

[B58] Dai J. Patzke C. Liakath-Ali K. Seigneur E. Sudhof T. C. (2021). GluD1 is a signal transduction device disguised as an ionotropic receptor. *Nature* 595 261–265. 10.1038/s41586-021-03661-6 34135511 PMC8776294

[B59] Dando O. McQueen J. Burr K. Kind P. C. Chandran S. Hardingham G. E. (2024). A comparison of basal and activity-dependent exon splicing in cortical-patterned neurons of human and mouse origin. *Front. Mol. Neurosci.* 17:1392408. 10.3389/fnmol.2024.1392408 39268251 PMC11390650

[B60] Davatolhagh M. F. Fuccillo M. V. (2021). Neurexin1α differentially regulates synaptic efficacy within striatal circuits. *Cell Rep.* 34:108773. 10.1016/j.celrep.2021.108773 33626349 PMC8071350

[B61] De Crescenzo F. Postorino V. Siracusano M. Riccioni A. Armando M. Curatolo P. (2019). Autistic symptoms in schizophrenia spectrum disorders: A systematic review and meta-analysis. *Front. Psychiatry* 10:78. 10.3389/fpsyt.2019.00078 30846948 PMC6393379

[B62] De Rubeis S. He X. Goldberg A. P. Poultney C. S. Samocha K. Cicek A. E. (2014). Synaptic, transcriptional and chromatin genes disrupted in autism. *Nature* 515 209–215. 10.1038/nature13772 25363760 PMC4402723

[B63] de Wit J. Sylwestrak E. O’Sullivan M. L. Otto S. Tiglio K. Savas J. N. (2009). LRRTM2 interacts with Neurexin1 and regulates excitatory synapse formation. *Neuron* 64 799–806. 10.1016/j.neuron.2009.12.019 20064388 PMC2829666

[B64] Dudanova I. Sedej S. Ahmad M. Masius H. Sargsyan V. Zhang W. (2006). Important contribution of alpha-neurexins to Ca2+-triggered exocytosis of secretory granules. *J. Neurosci.* 26 10599–10613. 10.1523/JNEUROSCI.1913-06.2006 17035546 PMC6674690

[B65] Dudanova I. Tabuchi K. Rohlmann A. Sudhof T. C. Missler M. (2007). Deletion of alpha-neurexins does not cause a major impairment of axonal pathfinding or synapse formation. *J. Comp. Neurol.* 502 261–274. 10.1002/cne.21305 17347997

[B66] Duong L. Klitten L. L. Moller R. S. Ingason A. Jakobsen K. D. Skjodt C. (2012). Mutations in NRXN1 in a family multiply affected with brain disorders: NRXN1 mutations and brain disorders. *Am. J. Med. Genet. B Neuropsychiatr. Genet.* 159B 354–358. 10.1002/ajmg.b.32036 22337556

[B67] Elegheert J. Cvetkovska V. Clayton A. J. Heroven C. Vennekens K. M. Smukowski S. N. (2017). Structural mechanism for modulation of synaptic neuroligin-neurexin signaling by MDGA proteins. *Neuron* 96 242–244. 10.1016/j.neuron.2017.09.011 28957672 PMC5625881

[B68] Eroglu C. (2009). The role of astrocyte-secreted matricellular proteins in central nervous system development and function. *J. Cell. Commun. Signal.* 3 167–176. 10.1007/s12079-009-0078-y 19904629 PMC2778595

[B69] Ervasti J. M. Campbell K. P. (1991). Membrane organization of the dystrophin-glycoprotein complex. *Cell* 66 1121–1131. 10.1016/0092-8674(91)90035-w 1913804

[B70] Esclassan F. Francois J. Phillips K. G. Loomis S. Gilmour G. (2015). Phenotypic characterization of nonsocial behavioral impairment in neurexin 1alpha knockout rats. *Behav. Neurosci.* 129 74–85. 10.1037/bne0000024 25420124

[B71] Etherton M. R. Blaiss C. A. Powell C. M. Sudhof T. C. (2009). Mouse neurexin-1alpha deletion causes correlated electrophysiological and behavioral changes consistent with cognitive impairments. *Proc. Natl. Acad. Sci. U S A.* 106 17998–18003. 10.1073/pnas.0910297106 19822762 PMC2764944

[B72] Falcao M. Monteiro P. Jacinto L. (2024). Tactile sensory processing deficits in genetic mouse models of autism spectrum disorder. *J. Neurochem.* 168 2105–2123. 10.1111/jnc.16135 38837765

[B73] Fan S. Gangwar S. P. Machius M. Rudenko G. (2021). Interplay between hevin, SPARC, and MDGAs: Modulators of neurexin-neuroligin transsynaptic bridges. *Structure* 29 664–678.e666. 10.1016/j.str.2021.01.003 33535026 PMC8254758

[B74] Farooqui A. A. Farooqui T. Panza F. Frisardi V. (2012). Metabolic syndrome as a risk factor for neurological disorders. *Cell. Mol. Life Sci.* 69 741–762. 10.1007/s00018-011-0840-1 21997383 PMC11115054

[B75] Felix R. Gurnett C. A. De Waard M. Campbell K. P. (1997). Dissection of functional domains of the voltage-dependent Ca2+ channel alpha2delta subunit. *J. Neurosci.* 17 6884–6891. 10.1523/JNEUROSCI.17-18-06884.1997 9278523 PMC6573258

[B76] Fell B. Eckrich S. Blum K. Eckrich T. Hecker D. Obermair G. J. (2016). alpha2delta2 controls the function and trans-synaptic coupling of Cav1.3 channels in mouse inner hair cells and is essential for normal hearing. *J. Neurosci.* 36 11024–11036. 10.1523/JNEUROSCI.3468-14.2016 27798183 PMC6705655

[B77] Feng J. Schroer R. Yan J. Song W. Yang C. Bockholt A. (2006). High frequency of neurexin 1beta signal peptide structural variants in patients with autism. *Neurosci. Lett.* 409 10–13. 10.1016/j.neulet.2006.08.017 17034946

[B78] Fernando M. B. Fan Y. Zhang Y. Tokolyi A. Murphy A. N. Kammourh S. (2025). Phenotypic complexities of rare heterozygous neurexin-1 deletions. *Nature* 642 710–720. 10.1038/s41586-025-08864-9 40205044

[B79] Flaherty E. Zhu S. Barretto N. Cheng E. Deans P. J. M. Fernando M. B. (2019). Neuronal impact of patient-specific aberrant NRXN1alpha splicing. *Nat. Genet.* 51 1679–1690. 10.1038/s41588-019-0539-z 31784728 PMC7451045

[B80] Fromer M. Pocklington A. J. Kavanagh D. H. Williams H. J. Dwyer S. Gormley P. (2014). De novo mutations in schizophrenia implicate synaptic networks. *Nature* 506 179–184. 10.1038/nature12929 24463507 PMC4237002

[B81] Fruh S. Romanos J. Panzanelli P. Burgisser D. Tyagarajan S. K. Campbell K. P. (2016). Neuronal dystroglycan is necessary for formation and maintenance of functional CCK-positive basket cell terminals on pyramidal cells. *J. Neurosci.* 36 10296–10313. 10.1523/JNEUROSCI.1823-16.2016 27707967 PMC6705590

[B82] Fu Y. Huang Z. J. (2010). Differential dynamics and activity-dependent regulation of alpha- and beta-neurexins at developing GABAergic synapses. *Proc. Natl. Acad. Sci. U S A.* 107 22699–22704. 10.1073/pnas.1011233108 21149722 PMC3012487

[B83] Fuccillo M. V. Pak C. (2021). Copy number variants in neurexin genes: Phenotypes and mechanisms. *Curr. Opin. Genet. Dev.* 68 64–70. 10.1016/j.gde.2021.02.010 33756113 PMC8491281

[B84] Fuccillo M. V. Foldy C. Gokce O. Rothwell P. E. Sun G. L. Malenka R. C. (2015). Single-cell mRNA profiling reveals cell-type-specific expression of neurexin isoforms. *Neuron* 87 326–340. 10.1016/j.neuron.2015.06.028 26182417 PMC4733560

[B85] Furlanis E. Traunmuller L. Fucile G. Scheiffele P. (2019). Landscape of ribosome-engaged transcript isoforms reveals extensive neuronal-cell-class-specific alternative splicing programs. *Nat. Neurosci.* 22 1709–1717. 10.1038/s41593-019-0465-5 31451803 PMC6763336

[B86] Gan K. J. Sudhof T. C. (2019). Specific factors in blood from young but not old mice directly promote synapse formation and NMDA-receptor recruitment. *Proc. Natl. Acad. Sci. U S A.* 116 12524–12533. 10.1073/pnas.1902672116 31160442 PMC6589664

[B87] Gan K. J. Sudhof T. C. (2020). SPARCL1 promotes excitatory but not inhibitory synapse formation and function independent of neurexins and neuroligins. *J. Neurosci.* 40 8088–8102. 10.1523/JNEUROSCI.0454-20.2020 32973045 PMC7574652

[B88] Ge L. Zhuo Y. Wu P. Liu Y. Qi L. Teng X. (2019). Olfactory ensheathing cells facilitate neurite sprouting and outgrowth by secreting high levels of hevin. *J. Chem. Neuroanat.* 104:101728. 10.1016/j.jchemneu.2019.101728 31783092

[B89] Geisler S. Schopf C. L. Stanika R. Kalb M. Campiglio M. Repetto D. (2019). Presynaptic alpha(2)delta-2 calcium channel subunits regulate postsynaptic GABA(A) receptor abundance and axonal wiring. *J. Neurosci.* 39 2581–2605. 10.1523/JNEUROSCI.2234-18.2019 30683685 PMC6445987

[B90] Gigliotti F. Giovannone F. Belli A. Sogos C. (2024). Atypical sensory processing in neurodevelopmental disorders: clinical phenotypes in preschool-aged children. *Children* 11:875. 10.3390/children11070875 39062324 PMC11276037

[B91] Gomez A. M. Traunmuller L. Scheiffele P. (2021). Neurexins: Molecular codes for shaping neuronal synapses. *Nat. Rev. Neurosci.* 22 137–151. 10.1038/s41583-020-00415-7 33420412 PMC7612283

[B92] Gongidi V. Ring C. Moody M. Brekken R. Sage E. H. Rakic P. (2004). SPARC-like 1 regulates the terminal phase of radial glia-guided migration in the cerebral cortex. *Neuron* 41 57–69. 10.1016/s0896-6273(03)00818-3 14715135

[B93] Grady R. M. Wozniak D. F. Ohlemiller K. K. Sanes J. R. (2006). Cerebellar synaptic defects and abnormal motor behavior in mice lacking alpha- and beta-dystrobrevin. *J. Neurosci.* 26 2841–2851. 10.1523/JNEUROSCI.4823-05.2006 16540561 PMC6673965

[B94] Grayton H. M. Missler M. Collier D. A. Fernandes C. (2013). Altered social behaviours in neurexin 1alpha knockout mice resemble core symptoms in neurodevelopmental disorders. *PLoS One* 8:e67114. 10.1371/journal.pone.0067114 23840597 PMC3696036

[B95] Grice D. E. Buxbaum J. D. (2006). The genetics of autism spectrum disorders. *Neuromolecular Med.* 8 451–460. 10.1385/NMM:8:4:451 17028369

[B96] Guzman C. Mohri K. Nakamura R. Miyake M. Tsuchiya Y. Tomii K. (2024). Neuronal and non-neuronal functions of the synaptic cell adhesion molecule neurexin in Nematostella vectensis. *Nat. Commun.* 15:6495. 10.1038/s41467-024-50818-8 39090098 PMC11294457

[B97] Haklai-Topper L. Soutschek J. Sabanay H. Scheel J. Hobert O. Peles E. (2011). The neurexin superfamily of Caenorhabditis elegans. *Gene Expr. Patterns* 11 144–150. 10.1016/j.gep.2010.10.008 21055481 PMC12414531

[B98] Hambrock H. O. Nitsche D. P. Hansen U. Bruckner P. Paulsson M. Maurer P. (2003). SC1/hevin. An extracellular calcium-modulated protein that binds collagen I. *J. Biol. Chem.* 278 11351–11358. 10.1074/jbc.M212291200 12538579

[B99] Hara Y. Balci-Hayta B. Yoshida-Moriguchi T. Kanagawa M. Beltran-Valero, de Bernabe D. (2011). A dystroglycan mutation associated with limb-girdle muscular dystrophy. *N. Engl. J. Med.* 364 939–946. 10.1056/NEJMoa1006939 21388311 PMC3071687

[B100] Harkin L. F. Lindsay S. J. Xu Y. Alzu’bi A. Ferrara A. Gullon E. A. (2017). Neurexins 1-3 each have a distinct pattern of expression in the early developing human cerebral cortex. *Cereb. Cortex* 27 216–232. 10.1093/cercor/bhw394 28013231 PMC5654756

[B101] Hata Y. Butz S. Sudhof T. C. (1996). CASK: A novel dlg/PSD95 homolog with an N-terminal calmodulin-dependent protein kinase domain identified by interaction with neurexins. *J. Neurosci.* 16 2488–2494. 10.1523/JNEUROSCI.16-08-02488.1996 8786425 PMC6578772

[B102] Hauser D. Behr K. Konno K. Schreiner D. Schmidt A. Watanabe M. (2022). Targeted proteoform mapping uncovers specific Neurexin-3 variants required for dendritic inhibition. *Neuron* 110 2094–2109.e2010. 10.1016/j.neuron.2022.04.017 35550065 PMC9275415

[B103] Hishimoto A. Liu Q. R. Drgon T. Pletnikova O. Walther D. Zhu X. G. (2007). Neurexin 3 polymorphisms are associated with alcohol dependence and altered expression of specific isoforms. *Hum. Mol. Genet.* 16 2880–2891. 10.1093/hmg/ddm247 17804423

[B104] Hohenester E. Maurer P. Timpl R. (1997). Crystal structure of a pair of follistatin-like and EF-hand calcium-binding domains in BM-40. *EMBO J.* 16 3778–3786. 10.1093/emboj/16.13.3778 9233787 PMC1170001

[B105] Holt K. H. Crosbie R. H. Venzke D. P. Campbell K. P. (2000). Biosynthesis of dystroglycan: processing of a precursor propeptide. *FEBS Lett.* 468 79–83. 10.1016/s0014-5793(00)01195-9 10683445

[B106] Hoppa M. B. Lana B. Margas W. Dolphin A. C. Ryan T. A. (2012). alpha2delta expression sets presynaptic calcium channel abundance and release probability. *Nature* 486 122–125. 10.1038/nature11033 22678293 PMC3376018

[B107] Hu X. Zhang J. Jin C. Mi W. Wang F. Ma W. (2013). Association study of NRXN3 polymorphisms with schizophrenia and risperidone-induced bodyweight gain in Chinese Han population. *Prog. Neuropsychopharmacol. Biol. Psychiatry* 43 197–202. 10.1016/j.pnpbp.2012.12.007 23306218

[B108] Hu Z. Xiao X. Zhang Z. Li M. (2019). Genetic insights and neurobiological implications from NRXN1 in neuropsychiatric disorders. *Mol. Psychiatry* 24 1400–1414. 10.1038/s41380-019-0438-9 31138894

[B109] Huang A. Y. Yu D. Davis L. K. Sul J. H. Tsetsos F. Ramensky V. (2017). Rare copy number variants in NRXN1 and CNTN6 increase risk for tourette syndrome. *Neuron* 94 1101–1111.e1107. 10.1016/j.neuron.2017.06.010 28641109 PMC5568251

[B110] Ibraghimov-Beskrovnaya O. Ervasti J. M. Leveille C. J. Slaughter C. A. Sernett S. W. Campbell K. P. (1992). Primary structure of dystrophin-associated glycoproteins linking dystrophin to the extracellular matrix. *Nature* 355 696–702. 10.1038/355696a0 1741056

[B111] Ichtchenko K. Hata Y. Nguyen T. Ullrich B. Missler M. Moomaw C. (1995). Neuroligin 1: A splice site-specific ligand for beta-neurexins. *Cell* 81 435–443. 10.1016/0092-8674(95)90396-8 7736595

[B112] Ikeda M. Aleksic B. Kirov G. Kinoshita Y. Yamanouchi Y. Kitajima T. (2010). Copy number variation in schizophrenia in the Japanese population. *Biol. Psychiatry* 67 283–286. 10.1016/j.biopsych.2009.08.034 19880096

[B113] Iossifov I. Ronemus M. Levy D. Wang Z. Hakker I. Rosenbaum J. (2012). De novo gene disruptions in children on the autistic spectrum. *Neuron* 74 285–299. 10.1016/j.neuron.2012.04.009 22542183 PMC3619976

[B114] Iwasaki S. Momiyama A. Uchitel O. D. Takahashi T. (2000). Developmental changes in calcium channel types mediating central synaptic transmission. *J. Neurosci.* 20 59–65. 10.1523/JNEUROSCI.20-01-00059.2000 10627581 PMC6774098

[B115] Jahncke J. N. Miller D. S. Krush M. Schnell E. Wright K. M. (2024). Inhibitory CCK+ basket synapse defects in mouse models of dystroglycanopathy. *Elife* 12:e87965. 10.7554/eLife.87965 38179984 PMC10942650

[B116] Jahncke J. N. Schnell E. Wright K. M. (2025). Distinct functional domains of Dystroglycan regulate inhibitory synapse formation and maintenance in cerebellar Purkinje cells. *Commun. Biol.* 8:878. 10.1038/s42003-025-08323-1 40473926 PMC12141699

[B117] Janz P. Bainier M. Marashli S. Schoenenberger P. Valencia M. Redondo R. L. (2022). Neurexin1alpha knockout rats display oscillatory abnormalities and sensory processing deficits back-translating key endophenotypes of psychiatric disorders. *Transl. Psychiatry* 12:455. 10.1038/s41398-022-02224-1 36307390 PMC9616904

[B118] Javitt D. C. (2009). Sensory processing in schizophrenia: Neither simple nor intact. *Schizophr. Bull.* 35 1059–1064. 10.1093/schbul/sbp110 19833806 PMC2762632

[B119] Jayakumar A. R. Apeksha A. Norenberg M. D. (2017). Role of matricellular proteins in disorders of the central nervous system. *Neurochem. Res.* 42 858–875. 10.1007/s11064-016-2088-5 27878658

[B120] Jenkins A. K. Paterson C. Wang Y. Hyde T. M. Kleinman J. E. Law A. J. (2016). Neurexin 1 (NRXN1) splice isoform expression during human neocortical development and aging. *Mol. Psychiatry* 21 701–706. 10.1038/mp.2015.107 26216298 PMC4731316

[B121] Jeong J. Pandey S. Li Y. Badger J. D. Lu W. Roche K. W. (2019). PSD-95 binding dynamically regulates NLGN1 trafficking and function. *Proc. Natl. Acad. Sci. U S A.* 116 12035–12044. 10.1073/pnas.1821775116 31138690 PMC6575593

[B122] Johnston I. G. Paladino T. Gurd J. W. Brown I. R. (1990). Molecular cloning of SC1: A putative brain extracellular matrix glycoprotein showing partial similarity to osteonectin/BM40/SPARC. *Neuron* 4 165–176. 10.1016/0896-6273(90)90452-l 1690015

[B123] Jones E. V. Bouvier D. S. (2014). Astrocyte-secreted matricellular proteins in CNS remodelling during development and disease. *Neural Plas* 2014:321209. 10.1155/2014/321209 24551460 PMC3914553

[B124] Kang M. H. Oh D. J. Rhee D. J. (2011). Effect of hevin deletion in mice and characterization in trabecular meshwork. *Invest. Ophthalmol. Vis. Sci.* 52 2187–2193. 10.1167/iovs.10-5428 21220554 PMC3080182

[B125] Kang Y. Xue J. Zheng J. Liang J. Cai C. Wang Y. (2022). Upregulation of Hevin contributes to postoperative pain hypersensitivity by inducing neurexin1beta/neuroligin1-mediated synaptic targeting of GluA1-containing AMPA receptors in rat dorsal horn. *Brain Res.* 1792:148004. 10.1016/j.brainres.2022.148004 35820448

[B126] Kang Y. Zhang X. Dobie F. Wu H. Craig A. M. (2008). Induction of GABAergic postsynaptic differentiation by alpha-neurexins. *J. Biol. Chem.* 283 2323–2334. 10.1074/jbc.M703957200 18006501 PMC2811689

[B127] Kasem E. Kurihara T. Tabuchi K. (2018). Neurexins and neuropsychiatric disorders. *Neurosci. Res.* 127 53–60. 10.1016/j.neures.2017.10.012 29221905

[B128] Kattenstroth G. Tantalaki E. Sudhof T. C. Gottmann K. Missler M. (2004). Postsynaptic N-methyl-D-aspartate receptor function requires alpha-neurexins. *Proc. Natl. Acad. Sci. U S A.* 101 2607–2612. 10.1073/pnas.0308626100 14983056 PMC356997

[B129] Kazdoba T. M. Leach P. T. Yang M. Silverman J. L. Solomon M. Crawley J. N. (2016). Translational mouse models of autism: Advancing toward pharmacological therapeutics. *Curr. Top. Behav. Neurosci.* 28 1–52. 10.1007/7854_2015_5003 27305922 PMC5116923

[B130] Khoja S. Haile M. T. Chen L. Y. (2023). Advances in neurexin studies and the emerging role of neurexin-2 in autism spectrum disorder. *Front. Mol. Neurosci.* 16:1125087. 10.3389/fnmol.2023.1125087 36923655 PMC10009110

[B131] Kight K. E. Argue K. J. Bumgardner J. G. Bardhi K. Waddell J. McCarthy M. M. (2021). Social behavior in prepubertal neurexin 1alpha deficient rats: A model of neurodevelopmental disorders. *Behav. Neurosci.* 135 782–803. 10.1037/bne0000482 34323517 PMC8649076

[B132] Kim H. G. Kishikawa S. Higgins A. W. Seong I. S. Donovan D. J. Shen Y. (2008). Disruption of neurexin 1 associated with autism spectrum disorder. *Am. J. Hum. Genet.* 82 199–207. 10.1016/j.ajhg.2007.09.011 18179900 PMC2253961

[B133] Kim J. H. Jung H. G. Kim A. Shim H. S. Hyeon S. J. Lee Y. S. (2021). Hevin-calcyon interaction promotes synaptic reorganization after brain injury. *Cell. Death Differ.* 28 2571–2588. 10.1038/s41418-021-00772-5 33753902 PMC8408247

[B134] Kirov G. Gumus D. Chen W. Norton N. Georgieva L. Sari M. (2008). Comparative genome hybridization suggests a role for NRXN1 and APBA2 in schizophrenia. *Hum. Mol. Genet.* 17 458–465. 10.1093/hmg/ddm323 17989066

[B135] Klatt O. Repetto D. Brockhaus J. Reissner C. El Khallouqi A. Rohlmann A. (2021). Endogenous beta-neurexins on axons and within synapses show regulated dynamic behavior. *Cell Rep.* 35:109266. 10.1016/j.celrep.2021.109266 34133920

[B136] Klei L. McClain L. L. Mahjani B. Panayidou K. De Rubeis S. Grahnat A. S. (2021). How rare and common risk variation jointly affect liability for autism spectrum disorder. *Mol. Autism* 12:66. 10.1186/s13229-021-00466-2 34615521 PMC8495987

[B137] Klugbauer N. Lacinova L. Marais E. Hobom M. Hofmann F. (1999). Molecular diversity of the calcium channel alpha2delta subunit. *J. Neurosci.* 19 684–691. 10.1523/JNEUROSCI.19-02-00684.1999 9880589 PMC6782206

[B138] Ko J. Fuccillo M. V. Malenka R. C. Sudhof T. C. (2009). LRRTM2 functions as a neurexin ligand in promoting excitatory synapse formation. *Neuron* 64 791–798. 10.1016/j.neuron.2009.12.012 20064387 PMC2829314

[B139] Kramer P. R. Hornung R. S. Umorin M. Benson M. D. Kinchington P. R. (2024). Neurexin 3 regulates synaptic connections between central amygdala neurons and excitable cells of the lateral parabrachial nucleus in rats with varicella zoster induced orofacial pain. *J. Pain Res.* 17 2311–2324. 10.2147/JPR.S441706 38974829 PMC11227312

[B140] Kramer P. R. Umorin M. Hornung R. Benson M. D. Kinchington P. R. (2022). Sex differences in the role of Neurexin 3alpha in zoster associated pain. *Front. Integr. Neurosci.* 16:915797. 10.3389/fnint.2022.915797 35875508 PMC9302461

[B141] Kucukdereli H. Allen N. J. Lee A. T. Feng A. Ozlu M. I. Conatser L. M. (2011). Control of excitatory CNS synaptogenesis by astrocyte-secreted proteins Hevin and SPARC. *Proc. Natl. Acad. Sci U S A* 108 E440–E449. 10.1073/pnas.1104977108 21788491 PMC3156217

[B142] Laarakker M. C. Reinders N. R. Bruining H. Ophoff R. A. Kas M. J. (2012). Sex-dependent novelty response in neurexin-1alpha mutant mice. *PLoS One* 7:e31503. 10.1371/journal.pone.0031503 22348092 PMC3278455

[B143] Lam M. Moslem M. Bryois J. Pronk R. J. Uhlin E. Ellstrom I. D. (2019). Single cell analysis of autism patient with bi-allelic NRXN1-alpha deletion reveals skewed fate choice in neural progenitors and impaired neuronal functionality. *Exp. Cell. Res.* 383:111469. 10.1016/j.yexcr.2019.06.014 31302032

[B144] Le A. D. Fu M. Carper A. Zegarowicz E. Kumar R. Zacharias G. (2025). Astrocyte modulation of synaptic plasticity mediated by activity-dependent sonic hedgehog signaling. *J. Neurosci.* 45:e1336242025. 10.1523/JNEUROSCI.1336-24.2025 39900499 PMC11905353

[B145] Levi S. Grady R. M. Henry M. D. Campbell K. P. Sanes J. R. Craig A. M. (2002). Dystroglycan is selectively associated with inhibitory GABAergic synapses but is dispensable for their differentiation. *J. Neurosci.* 22 4274–4285. 10.1523/JNEUROSCI.22-11-04274.2002 12040032 PMC6758826

[B146] Liu H. Huang X. Xu J. Mao H. Li Y. Ren K. (2021). Dissection of the relationship between anxiety and stereotyped self-grooming using the Shank3B mutant autistic model, acute stress model and chronic pain model. *Neurobiol. Stress* 15:100417. 10.1016/j.ynstr.2021.100417 34815987 PMC8591549

[B147] Liu S. Kuja-Halkola R. Larsson H. Lichtenstein P. Ludvigsson J. F. Svensson A. M. (2021). Neurodevelopmental disorders, glycemic control, and diabetic complications in type 1 diabetes: A nationwide cohort study. *J. Clin. Endocrinol. Metab.* 106 e4459–e4470. 10.1210/clinem/dgab467 34171098 PMC8530713

[B148] Liu X. Ying G. Wang W. Dong J. Wang Y. Ni Z. (2005). Entorhinal deafferentation induces upregulation of SPARC in the mouse hippocampus. *Brain Res. Mol. Brain Res.* 141 58–65. 10.1016/j.molbrainres.2005.08.003 16137785

[B149] Liu Y. Hu Z. Xun G. Peng Y. Lu L. Xu X. (2012). Mutation analysis of the NRXN1 gene in a Chinese autism cohort. *J. Psychiatr. Res.* 46 630–634. 10.1016/j.jpsychires.2011.10.015 22405623

[B150] Lively S. Brown I. R. (2007). Analysis of the extracellular matrix protein SC1 during reactive gliosis in the rat lithium-pilocarpine seizure model. *Brain Res.* 1163 1–9. 10.1016/j.brainres.2007.05.052 17628511

[B151] Lively S. Brown I. R. (2008). Localization of the extracellular matrix protein SC1 coincides with synaptogenesis during rat postnatal development. *Neurochem. Res.* 33 1692–1700. 10.1007/s11064-008-9606-z 18335312

[B152] Lively S. Ringuette M. J. Brown I. R. (2007). Localization of the extracellular matrix protein SC1 to synapses in the adult rat brain. *Neurochem. Res.* 32 65–71. 10.1007/s11064-006-9226-4 17151913

[B153] Lloyd B. A. Han Y. Roth R. Zhang B. Aoto J. (2023). Neurexin-3 subsynaptic densities are spatially distinct from Neurexin-1 and essential for excitatory synapse nanoscale organization in the hippocampus. *Nat. Commun.* 14:4706. 10.1038/s41467-023-40419-2 37543682 PMC10404257

[B154] Lu H. Zuo L. Roddick K. M. Zhang P. Oku S. Garden J. (2023). Alternative splicing and heparan sulfation converge on neurexin-1 to control glutamatergic transmission and autism-related behaviors. *Cell. Rep.* 42:112714. 10.1016/j.celrep.2023.112714 37384525

[B155] Lukacsovich D. Winterer J. Que L. Luo W. Lukacsovich T. Foldy C. (2019). Single-Cell RNA-Seq reveals developmental origins and ontogenetic stability of neurexin alternative splicing profiles. *Cell. Rep.* 27 3752–3759 e3754. 10.1016/j.celrep.2019.05.090 31242409

[B156] Luo F. Sclip A. Jiang M. Sudhof T. C. (2020). Neurexins cluster Ca(2+) channels within the presynaptic active zone. *EMBO J.* 39:e103208. 10.15252/embj.2019103208 32134527 PMC7110102

[B157] Luo F. Sclip A. Merrill S. Sudhof T. C. (2021). Neurexins regulate presynaptic GABA(B)-receptors at central synapses. *Nat. Commun.* 12:2380. 10.1038/s41467-021-22753-5 33888718 PMC8062527

[B158] Marashli S. Janz P. Redondo R. L. (2024). Age-dependent deficits of auditory brainstem responses in juvenile Neurexin1alpha knockout rats. *Sci. Rep.* 14:22614. 10.1038/s41598-024-73920-9 39349722 PMC11443144

[B159] Marco E. J. Hinkley L. B. Hill S. S. Nagarajan S. S. (2011). Sensory processing in autism: A review of neurophysiologic findings. *Pediatr. Res.* 69 48R–54R. 10.1203/PDR.0b013e3182130c54 21289533 PMC3086654

[B160] McKinnon P. J. Margolskee R. F. (1996). SC1: A marker for astrocytes in the adult rodent brain is upregulated during reactive astrocytosis. *Brain Res.* 709 27–36. 10.1016/0006-8993(95)01224-9 8869553

[B161] McKinnon P. J. McLaughlin S. K. Kapsetaki M. Margolskee R. F. (2000). Extracellular matrix-associated protein Sc1 is not essential for mouse development. *Mol. Cell. Biol.* 20 656–660. 10.1128/MCB.20.2.656-660.2000 10611244 PMC85160

[B162] Mendis D. B. Ivy G. O. Brown I. R. (1996). SC1, a brain extracellular matrix glycoprotein related to SPARC and follistatin, is expressed by rat cerebellar astrocytes following injury and during development. *Brain Res.* 730 95–106. 10.1016/0006-8993(96)00440-4 8883893

[B163] Mendis D. B. Ivy G. O. Brown I. R. (2000). Induction of SC1 mRNA encoding a brain extracellular matrix glycoprotein related to SPARC following lesioning of the adult rat forebrain. *Neurochem. Res.* 25 1637–1644. 10.1023/a:1026626805612 11152393

[B164] Mendis D. B. Malaval L. Brown I. R. (1995). SPARC, an extracellular matrix glycoprotein containing the follistatin module, is expressed by astrocytes in synaptic enriched regions of the adult brain. *Brain Res.* 676 69–79. 10.1016/0006-8993(95)00101-u 7796180

[B165] Meng X. McGraw C. M. Wang W. Jing J. Yeh S. Y. Wang L. (2019). Neurexophilin4 is a selectively expressed alpha-neurexin ligand that modulates specific cerebellar synapses and motor functions. *Elife* 8:e46773. 10.7554/eLife.46773 31524598 PMC6763262

[B166] Miller D. S. Wright K. M. (2021). Neuronal Dystroglycan regulates postnatal development of CCK/cannabinoid receptor-1 interneurons. *Neural Dev.* 16:4. 10.1186/s13064-021-00153-1 34362433 PMC8349015

[B167] Miller M. T. Mileni M. Comoletti D. Stevens R. C. Harel M. Taylor P. (2011). The crystal structure of the alpha-neurexin-1 extracellular region reveals a hinge point for mediating synaptic adhesion and function. *Structure* 19 767–778. 10.1016/j.str.2011.03.011 21620717 PMC3279696

[B168] Missler M. Sudhof T. C. (1998). Neurexophilins form a conserved family of neuropeptide-like glycoproteins. *J. Neurosci.* 18 3630–3638. 10.1523/JNEUROSCI.18-10-03630.1998 9570794 PMC6793134

[B169] Missler M. Hammer R. E. Sudhof T. C. (1998). Neurexophilin binding to alpha-neurexins. A single LNS domain functions as an independently folding ligand-binding unit. *J. Biol. Chem.* 273 34716–34723. 10.1074/jbc.273.52.34716 9856994

[B170] Missler M. Zhang W. Rohlmann A. Kattenstroth G. Hammer R. E. Gottmann K. (2003). Alpha-neurexins couple Ca2+ channels to synaptic vesicle exocytosis. *Nature* 423 939–948. 10.1038/nature01755 12827191

[B171] Miyazaki T. Morimoto-Tomita M. Berthoux C. Konno K. Noam Y. Yamasaki T. (2021). Excitatory and inhibitory receptors utilize distinct post- and trans-synaptic mechanisms in vivo. *Elife* 10:59613. 10.7554/eLife.59613 34658339 PMC8550753

[B172] Mongredien R. Erdozain A. M. Dumas S. Cutando L. Del Moral A. N. Puighermanal E. (2019). Cartography of hevin-expressing cells in the adult brain reveals prominent expression in astrocytes and parvalbumin neurons. *Brain Struct. Funct.* 224 1219–1244. 10.1007/s00429-019-01831-x 30656447

[B173] Moons T. De Hert M. Gellens E. Gielen L. Sweers K. Jacqmaert S. (2016). Genetic evaluation of schizophrenia using the illumina humanexome chip. *PLoS One* 11:e0150464. 10.1371/journal.pone.0150464 27028512 PMC4814136

[B174] Mosedale M. Egodage S. Calma R. C. Chi N. W. Chessler S. D. (2012). Neurexin-1alpha contributes to insulin-containing secretory granule docking. *J. Biol. Chem.* 287 6350–6361. 10.1074/jbc.M111.299081 22235116 PMC3307300

[B175] Muller C. S. Haupt A. Bildl W. Schindler J. Knaus H. G. Meissner M. (2010). Quantitative proteomics of the Cav2 channel nano-environments in the mammalian brain. *Proc. Natl. Acad. Sci. U S A.* 107 14950–14957. 10.1073/pnas.1005940107 20668236 PMC2930569

[B176] Nag A. Bochukova E. G. Kremeyer B. Campbell D. D. Muller H. Valencia-Duarte A. V. (2013). CNV analysis in Tourette syndrome implicates large genomic rearrangements in COL8A1 and NRXN1. *PLoS One* 8:e59061. 10.1371/journal.pone.0059061 23533600 PMC3606459

[B177] Nakamura Y. Harada H. Kamasawa N. Matsui K. Rothman J. S. Shigemoto R. (2015). Nanoscale distribution of presynaptic Ca(2+) channels and its impact on vesicular release during development. *Neuron* 85 145–158. 10.1016/j.neuron.2014.11.019 25533484 PMC4305191

[B178] Neupert C. Schneider R. Klatt O. Reissner C. Repetto D. Biermann B. (2015). Regulated dynamic trafficking of neurexins inside and outside of synaptic terminals. *J. Neurosci.* 35 13629–13647. 10.1523/JNEUROSCI.4041-14.2015 26446217 PMC6605384

[B179] Nickolls A. R. Bonnemann C. G. (2018). The roles of dystroglycan in the nervous system: Insights from animal models of muscular dystrophy. *Dis. Model Mech.* 11:dmm035931. 10.1242/dmm.035931 30578246 PMC6307911

[B180] Nozawa K. Sogabe T. Hayashi A. Motohashi J. Miura E. Arai I. (2022). In vivo nanoscopic landscape of neurexin ligands underlying anterograde synapse specification. *Neuron* 110 3168–3185 e3168. 10.1016/j.neuron.2022.07.027 36007521

[B181] Nunez-delMoral A. Bianchi P. C. Brocos-Mosquera I. Anesio A. Palombo P. Camarini R. (2023). The matricellular protein hevin is involved in alcohol use disorder. *Biomolecules* 13:234. 10.3390/biom13020234 36830603 PMC9953008

[B182] Nunez-delMoral A. Brocos-Mosquera I. Vialou V. Callado L. F. Erdozain A. M. (2021). Characterization of hevin (SPARCL1) immunoreactivity in postmortem human brain homogenates. *Neuroscience* 467 91–109. 10.1016/j.neuroscience.2021.05.017 34033869

[B183] Nussbaum J. Xu Q. Payne T. J. Ma J. Z. Huang W. Gelernter J. (2008). Significant association of the neurexin-1 gene (NRXN1) with nicotine dependence in European- and African-American smokers. *Hum. Mol. Genet.* 17 1569–1577. 10.1093/hmg/ddn044 18270208 PMC2902291

[B184] Ostergaard F. G. Kas M. J. H. (2025). Seven unique frequency profiles for scoring vigilance states in preclinical electrophysiological data. *Front. Neurosci.* 19:1488709. 10.3389/fnins.2025.1488709 40370661 PMC12075235

[B185] Pak C. Danko T. Mirabella V. R. Wang J. Liu Y. Vangipuram M. (2021). Cross-platform validation of neurotransmitter release impairments in schizophrenia patient-derived NRXN1-mutant neurons. *Proc. Natl. Acad. Sci. U S A.* 118:e2025598118. 10.1073/pnas.2025598118 34035170 PMC8179243

[B186] Pak C. Danko T. Zhang Y. Aoto J. Anderson G. Maxeiner S. (2015). Human neuropsychiatric disease modeling using conditional deletion reveals synaptic transmission defects caused by heterozygous mutations in NRXN1. *Cell Stem Cell.* 17 316–328. 10.1016/j.stem.2015.07.017 26279266 PMC4560990

[B187] Penninx B. Lange S. M. M. (2018). Metabolic syndrome in psychiatric patients: Overview, mechanisms, and implications. *Dialogues Clin. Neurosci.* 20 63–73. 10.31887/DCNS.2018.20.1/bpenninx 29946213 PMC6016046

[B188] Perez-Palma E. Helbig I. Klein K. M. Anttila V. Horn H. Reinthaler E. M. (2017). Heterogeneous contribution of microdeletions in the development of common generalised and focal epilepsies. *J. Med. Genet.* 54 598–606. 10.1136/jmedgenet-2016-104495 28756411 PMC5574393

[B189] Pervolaraki E. Tyson A. L. Pibiri F. Poulter S. L. Reichelt A. C. Rodgers R. J. (2019). The within-subject application of diffusion tensor MRI and CLARITY reveals brain structural changes in Nrxn2 deletion mice. *Mol. Autism* 10:8. 10.1186/s13229-019-0261-9 30858964 PMC6394023

[B190] Petrenko A. G. Ullrich B. Missler M. Krasnoperov V. Rosahl T. W. Sudhof T. C. (1996). Structure and evolution of neurexophilin. *J. Neurosci.* 16 4360–4369. 10.1523/JNEUROSCI.16-14-04360.1996 8699246 PMC6578849

[B191] Piek J. P. Dyck M. J. (2004). Sensory-motor deficits in children with developmental coordination disorder, attention deficit hyperactivity disorder and autistic disorder. *Hum. Mov. Sci.* 23 475–488. 10.1016/j.humov.2004.08.019 15541530

[B192] Pippucci T. Parmeggiani A. Palombo F. Maresca A. Angius A. Crisponi L. (2013). A novel null homozygous mutation confirms CACNA2D2 as a gene mutated in epileptic encephalopathy. *PLoS One* 8:e82154. 10.1371/journal.pone.0082154 24358150 PMC3864908

[B193] Poulopoulos A. Aramuni G. Meyer G. Soykan T. Hoon M. Papadopoulos T. (2009). Neuroligin 2 drives postsynaptic assembly at perisomatic inhibitory synapses through gephyrin and collybistin. *Neuron* 63 628–642. 10.1016/j.neuron.2009.08.023 19755106

[B194] Pribiag H. Peng H. Shah W. A. Stellwagen D. Carbonetto S. (2014). Dystroglycan mediates homeostatic synaptic plasticity at GABAergic synapses. *Proc. Natl. Acad. Sci. U S A.* 111 6810–6815. 10.1073/pnas.1321774111 24753587 PMC4020085

[B195] Purcell S. M. Moran J. L. Fromer M. Ruderfer D. Solovieff N. Roussos P. (2014). A polygenic burden of rare disruptive mutations in schizophrenia. *Nature* 506 185–190. 10.1038/nature12975 24463508 PMC4136494

[B196] Purisai M. G. Sands S. A. Davis T. D. Price J. L. Chronwall B. M. (2005). GABAB receptor subunit mRNAs are differentially regulated in pituitary melanotropes during development and detection of functioning receptors coincides with completion of innervation. *Int. J. Dev. Neurosci.* 23 315–326. 10.1016/j.ijdevneu.2005.01.005 15927755

[B197] Puschel A. W. Betz H. (1995). Neurexins are differentially expressed in the embryonic nervous system of mice. *J. Neurosci.* 15 2849–2856. 10.1523/JNEUROSCI.15-04-02849.1995 7722633 PMC6577774

[B198] Reichelt A. C. Rodgers R. J. Clapcote S. J. (2012). The role of neurexins in schizophrenia and autistic spectrum disorder. *Neuropharmacology* 62 1519–1526. 10.1016/j.neuropharm.2011.01.024 21262241

[B199] Reissner C. Missler M. (2011). Unveiled alpha-neurexins take center stage. *Structure* 19 749–750. 10.1016/j.str.2011.05.005 21645846

[B200] Reissner C. Runkel F. Missler M. (2013). Neurexins. *Genome Biol.* 14:213. 10.1186/gb-2013-14-9-213 24083347 PMC4056431

[B201] Reissner C. Stahn J. Breuer D. Klose M. Pohlentz G. Mormann M. (2014). Dystroglycan binding to alpha-neurexin competes with neurexophilin-1 and neuroligin in the brain. *J. Biol. Chem.* 289 27585–27603. 10.1074/jbc.M114.595413 25157101 PMC4183798

[B202] Ribeiro L. F. Verpoort B. Nys J. Vennekens K. M. Wierda K. D. de Wit J. (2019). SorCS1-mediated sorting in dendrites maintains neurexin axonal surface polarization required for synaptic function. *PLoS Biol.* 17:e3000466. 10.1371/journal.pbio.3000466 31658245 PMC6837583

[B203] Risher W. C. Patel S. Kim I. H. Uezu A. Bhagat S. Wilton D. K. (2014). Astrocytes refine cortical connectivity at dendritic spines. *Elife* 3:e04047. 10.7554/eLife.04047 25517933 PMC4286724

[B204] Rochtus A. M. Trowbridge S. Goldstein R. D. Sheidley B. R. Prabhu S. P. Haynes R. (2019). Mutations in NRXN1 and NRXN2 in a patient with early-onset epileptic encephalopathy and respiratory depression. *Cold Spring Harb. Mol. Case Stud.* 5:a003442. 10.1101/mcs.a003442 30709877 PMC6371743

[B205] Rujescu D. Ingason A. Cichon S. Pietilainen O. P. Barnes M. R. Toulopoulou T. (2009). Disruption of the neurexin 1 gene is associated with schizophrenia. *Hum. Mol. Genet.* 18 988–996. 10.1093/hmg/ddn351 18945720 PMC2695245

[B206] Schaaf C. P. Boone P. M. Sampath S. Williams C. Bader P. I. Mueller J. M. (2012). Phenotypic spectrum and genotype-phenotype correlations of NRXN1 exon deletions. *Eur. J. Hum. Genet.* 20 1240–1247. 10.1038/ejhg.2012.95 22617343 PMC3499754

[B207] Schizophrenia Working Group of the Psychiatric Genomics. (2014). Biological insights from 108 schizophrenia-associated genetic loci. *Nature* 511 421–427. 10.1038/nature13595 25056061 PMC4112379

[B208] Schneider R. Hosy E. Kohl J. Klueva J. Choquet D. Thomas U. (2015). Mobility of calcium channels in the presynaptic membrane. *Neuron* 86 672–679. 10.1016/j.neuron.2015.03.050 25892305

[B209] Schreiner D. Nguyen T. M. Russo G. Heber S. Patrignani A. Ahrne E. (2014). Targeted combinatorial alternative splicing generates brain region-specific repertoires of neurexins. *Neuron* 84 386–398. 10.1016/j.neuron.2014.09.011 25284007

[B210] Schreiner D. Simicevic J. Ahrne E. Schmidt A. Scheiffele P. (2015). Quantitative isoform-profiling of highly diversified recognition molecules. *Elife* 4:e07794. 10.7554/eLife.07794 25985086 PMC4489214

[B211] Sebastian R. Jin K. Pavon N. Bansal R. Potter A. Song Y. (2023). Schizophrenia-associated NRXN1 deletions induce developmental-timing- and cell-type-specific vulnerabilities in human brain organoids. *Nat. Commun.* 14:3770. 10.1038/s41467-023-39420-6 37355690 PMC10290702

[B212] Seddighi S. Varma V. R. An Y. Varma S. Beason-Held L. L. Tanaka T. (2018). SPARCL1 accelerates symptom onset in Alzheimer’s disease and influences brain structure and function during aging. *J. Alzheimers Dis.* 61 401–414. 10.3233/JAD-170557 29154276 PMC5934753

[B213] Shafer R. L. Wang Z. Bartolotti J. Mosconi M. W. (2021). Visual and somatosensory feedback mechanisms of precision manual motor control in autism spectrum disorder. *J. Neurodev. Disord.* 13:32. 10.1186/s11689-021-09381-2 34496766 PMC8427856

[B214] Shah D. P. Joshi M. Shedaliya U. Krishnakumar A. (2023). Recurrent hypoglycemia dampens functional regulation mediated via Neurexin-1, Neuroligin-2 and Mint-1 docking proteins: Intensified complications during diabetes. *Cell. Signal.* 104:110582. 10.1016/j.cellsig.2022.110582 36587752

[B215] Shiwaku H. Katayama S. Gao M. Kondo K. Nakano Y. Motokawa Y. (2023). Analyzing schizophrenia-related phenotypes in mice caused by autoantibodies against NRXN1alpha in schizophrenia. *Brain Behav. Immun.* 111 32–45. 10.1016/j.bbi.2023.03.028 37004758

[B216] Silverman J. L. Yang M. Lord C. Crawley J. N. (2010). Behavioural phenotyping assays for mouse models of autism. *Nat. Rev. Neurosci.* 11 490–502. 10.1038/nrn2851 20559336 PMC3087436

[B217] Simmons D. H. Titley H. K. Hansel C. Mason P. (2021). Behavioral tests for mouse models of autism: An argument for the inclusion of cerebellum-controlled motor behaviors. *Neuroscience* 462 303–319. 10.1016/j.neuroscience.2020.05.010 32417339

[B218] Singh S. K. Stogsdill J. A. Pulimood N. S. Dingsdale H. Kim Y. H. Pilaz L. J. (2016). Astrocytes assemble thalamocortical synapses by bridging NRX1alpha and NL1 via Hevin. *Cell* 164 183–196. 10.1016/j.cell.2015.11.034 26771491 PMC4715262

[B219] Sons M. S. Busche N. Strenzke N. Moser T. Ernsberger U. Mooren F. C. (2006). alpha-Neurexins are required for efficient transmitter release and synaptic homeostasis at the mouse neuromuscular junction. *Neuroscience* 138 433–446. 10.1016/j.neuroscience.2005.11.040 16406382

[B220] Sterky F. H. Trotter J. H. Lee S. J. Recktenwald C. V. Du X. Zhou B. (2017). Carbonic anhydrase-related protein CA10 is an evolutionarily conserved pan-neurexin ligand. *Proc. Natl. Acad. Sci. U S A.* 114 E1253–E1262. 10.1073/pnas.1621321114 28154140 PMC5320979

[B221] Stoltenberg S. F. Lehmann M. K. Christ C. C. Hersrud S. L. Davies G. E. (2011). Associations among types of impulsivity, substance use problems and neurexin-3 polymorphisms. *Drug Alcohol. Depend.* 119 e31–e38. 10.1016/j.drugalcdep.2011.05.025 21676558 PMC3254149

[B222] Strunz M. Jarrell J. T. Cohen D. S. Rosin E. R. Vanderburg C. R. Huang X. (2019). Modulation of SPARC/Hevin proteins in Alzheimer’s disease brain injury. *J. Alzheimers Dis.* 68 695–710. 10.3233/JAD-181032 30883351 PMC6481539

[B223] Suckow A. T. Comoletti D. Waldrop M. A. Mosedale M. Egodage S. Taylor P. (2008). Expression of neurexin, neuroligin, and their cytoplasmic binding partners in the pancreatic beta-cells and the involvement of neuroligin in insulin secretion. *Endocrinology* 149 6006–6017. 10.1210/en.2008-0274 18755801 PMC2613060

[B224] Suckow A. T. Zhang C. Egodage S. Comoletti D. Taylor P. Miller M. T. (2012). Transcellular neuroligin-2 interactions enhance insulin secretion and are integral to pancreatic beta cell function. *J. Biol. Chem.* 287 19816–19826. 10.1074/jbc.M111.280537 22528485 PMC3370167

[B225] Sudhof T. C. (2017). Synaptic neurexin complexes: A molecular code for the logic of neural circuits. *Cell* 171 745–769. 10.1016/j.cell.2017.10.024 29100073 PMC5694349

[B226] Sugita S. Saito F. Tang J. Satz J. Campbell K. Sudhof T. C. (2001). A stoichiometric complex of neurexins and dystroglycan in brain. *J. Cell. Biol.* 154 435–445. 10.1083/jcb.200105003 11470830 PMC2150755

[B227] Sundaram S. K. Huq A. M. Wilson B. J. Chugani H. T. (2010). Tourette syndrome is associated with recurrent exonic copy number variants. *Neurology* 74 1583–1590. 10.1212/WNL.0b013e3181e0f147 20427753 PMC2876824

[B228] Tabuchi K. Sudhof T. C. (2002). Structure and evolution of neurexin genes: Insight into the mechanism of alternative splicing. *Genomics* 79 849–859. 10.1006/geno.2002.6780 12036300

[B229] Taketomi T. Tsuruta F. (2023). Mutations in Hevin/Sparcl1 and risk of autism spectrum disorder. *Neural Regen. Res.* 18 1499–1500. 10.4103/1673-5374.361543 36571352 PMC10075111

[B230] Taketomi T. Yasuda T. Morita R. Kim J. Shigeta Y. Eroglu C. (2022). Autism-associated mutation in Hevin/Sparcl1 induces endoplasmic reticulum stress through structural instability. *Sci. Rep.* 12:11891. 10.1038/s41598-022-15784-5 35831437 PMC9279342

[B231] Tan R. L. Sciandra F. Hubner W. Bozzi M. Reimann J. Schoch S. (2024). The missense mutation C667F in murine beta-dystroglycan causes embryonic lethality, myopathy and blood-brain barrier destabilization. *Dis. Model Mech.* 17:dmm.050594. 10.1242/dmm.050594 38616731 PMC11212641

[B232] Tanabe Y. Naito Y. Vasuta C. Lee A. K. Soumounou Y. Linhoff M. W. (2017). IgSF21 promotes differentiation of inhibitory synapses via binding to neurexin2alpha. *Nat. Commun.* 8:408. 10.1038/s41467-017-00333-w 28864826 PMC5581337

[B233] Tanaka H. Miyazaki N. Matoba K. Nogi T. Iwasaki K. Takagi J. (2012). Higher-order architecture of cell adhesion mediated by polymorphic synaptic adhesion molecules neurexin and neuroligin. *Cell. Rep.* 2 101–110. 10.1016/j.celrep.2012.06.009 22840401

[B234] Tanaka H. Nogi T. Yasui N. Iwasaki K. Takagi J. (2011). Structural basis for variant-specific neuroligin-binding by alpha-neurexin. *PLoS One* 6:e19411. 10.1371/journal.pone.0019411 21552542 PMC3084293

[B235] Taniguchi H. Gollan L. Scholl F. G. Mahadomrongkul V. Dobler E. Limthong N. (2007). Silencing of neuroligin function by postsynaptic neurexins. *J. Neurosci.* 27 2815–2824. 10.1523/JNEUROSCI.0032-07.2007 17360903 PMC2839889

[B236] Tian M. Jacobson C. Gee S. H. Campbell K. P. Carbonetto S. Jucker M. (1996). Dystroglycan in the cerebellum is a laminin alpha 2-chain binding protein at the glial-vascular interface and is expressed in Purkinje cells. *Eur. J. Neurosci.* 8 2739–2747. 10.1111/j.1460-9568.1996.tb01568.x 8996823

[B237] Tong X. J. Lopez-Soto E. J. Li L. Liu H. Nedelcu D. Lipscombe D. (2017). Retrograde synaptic inhibition is mediated by alpha-neurexin binding to the alpha2delta subunits of N-type calcium channels. *Neuron* 95 326–340 e325. 10.1016/j.neuron.2017.06.018 28669545 PMC5548138

[B238] Traynelis S. F. Wollmuth L. P. McBain C. J. Menniti F. S. Vance K. M. Ogden K. K. (2010). Glutamate receptor ion channels: Structure, regulation, and function. *Pharmacol. Rev.* 62 405–496. 10.1124/pr.109.002451 20716669 PMC2964903

[B239] Treutlein B. Gokce O. Quake S. R. Sudhof T. C. (2014). Cartography of neurexin alternative splicing mapped by single-molecule long-read mRNA sequencing. *Proc. Natl. Acad. Sci. U S A.* 111 E1291–E1299. 10.1073/pnas.1403244111 24639501 PMC3977267

[B240] Trifu S. C. Kohn B. Vlasie A. Patrichi B. E. (2020). Genetics of schizophrenia (Review). *Exp. Ther. Med.* 20 3462–3468. 10.3892/etm.2020.8973 32905096 PMC7465115

[B241] Tromp A. Mowry B. Giacomotto J. (2021). Neurexins in autism and schizophrenia-a review of patient mutations, mouse models and potential future directions. *Mol. Psychiatry* 26 747–760. 10.1038/s41380-020-00944-8 33191396

[B242] Trotter J. H. Hao J. Maxeiner S. Tsetsenis T. Liu Z. Zhuang X. (2019). Synaptic neurexin-1 assembles into dynamically regulated active zone nanoclusters. *J. Cell. Biol.* 218 2677–2698. 10.1083/jcb.201812076 31262725 PMC6683742

[B243] Trotter J. H. Wang C. Y. Zhou P. Nakahara G. Sudhof T. C. (2023). A combinatorial code of neurexin-3 alternative splicing controls inhibitory synapses via a trans-synaptic dystroglycan signaling loop. *Nat. Commun.* 14:1771. 10.1038/s41467-023-36872-8 36997523 PMC10063607

[B244] Uchigashima M. Cheung A. Suh J. Watanabe M. Futai K. (2019). Differential expression of neurexin genes in the mouse brain. *J. Comp. Neurol.* 527 1940–1965. 10.1002/cne.24664 30761534 PMC6592846

[B245] Uchigashima M. Konno K. Demchak E. Cheung A. Watanabe T. Keener D. G. (2020). Specific Neuroligin3-alphaNeurexin1 signaling regulates GABAergic synaptic function in mouse hippocampus. *Elife* 9:e59545. 10.7554/eLife.59545 33355091 PMC7758064

[B246] Uemura T. Lee S. J. Yasumura M. Takeuchi T. Yoshida T. Ra M. (2010). Trans-synaptic interaction of GluRdelta2 and Neurexin through Cbln1 mediates synapse formation in the cerebellum. *Cell* 141 1068–1079. 10.1016/j.cell.2010.04.035 20537373

[B247] Ullrich B. Ushkaryov Y. A. Sudhof T. C. (1995). Cartography of neurexins: More than 1000 isoforms generated by alternative splicing and expressed in distinct subsets of neurons. *Neuron* 14 497–507. 10.1016/0896-6273(95)90306-2 7695896

[B248] Ushkaryov Y. A. Sudhof T. C. (1993). Neurexin III alpha: extensive alternative splicing generates membrane-bound and soluble forms. *Proc. Natl. Acad. Sci. U S A.* 90 6410–6414. 10.1073/pnas.90.14.6410 8341647 PMC46941

[B249] Ushkaryov Y. A. Hata Y. Ichtchenko K. Moomaw C. Afendis S. Slaughter C. A. (1994). Conserved domain structure of beta-neurexins. Unusual cleaved signal sequences in receptor-like neuronal cell-surface proteins. *J. Biol. Chem.* 269 11987–11992. 10.1016/S0021-9258(17)32671-6 8163501

[B250] Ushkaryov Y. A. Petrenko A. G. Geppert M. Sudhof T. C. (1992). Neurexins: synaptic cell surface proteins related to the alpha-latrotoxin receptor and laminin. *Science* 257 50–56. 10.1126/science.1621094 1621094

[B251] Vaags A. K. Lionel A. C. Sato D. Goodenberger M. Stein Q. P. Curran S. (2012). Rare deletions at the neurexin 3 locus in autism spectrum disorder. *Am. J. Hum. Genet.* 90 133–141. 10.1016/j.ajhg.2011.11.025 22209245 PMC3257896

[B252] Valence S. Cochet E. Rougeot C. Garel C. Chantot-Bastaraud S. Lainey E. (2019). Exome sequencing in congenital ataxia identifies two new candidate genes and highlights a pathophysiological link between some congenital ataxias and early infantile epileptic encephalopathies. *Genet. Med.* 21 553–563. 10.1038/s41436-018-0089-2 29997391

[B253] Vincent A. J. Lau P. W. Roskams A. J. (2008). SPARC is expressed by macroglia and microglia in the developing and mature nervous system. *Dev. Dyn.* 237 1449–1462. 10.1002/dvdy.21495 18366138

[B254] Wallingford J. Scott A. L. Rodrigues K. Doering L. C. (2017). Altered developmental expression of the astrocyte-secreted factors hevin and SPARC in the fragile X mouse model. *Front. Mol. Neurosci.* 10:268. 10.3389/fnmol.2017.00268 28900386 PMC5581809

[B255] Wang J. Gong J. Li L. Chen Y. Liu L. Gu H. (2018). Neurexin gene family variants as risk factors for autism spectrum disorder. *Autism Res.* 11 37–43. 10.1002/aur.1881 29045040

[B256] Wang K. Zhang H. Ma D. Bucan M. Glessner J. T. Abrahams B. S. (2009). Common genetic variants on 5p14.1 associate with autism spectrum disorders. *Nature* 459 528–533. 10.1038/nature07999 19404256 PMC2943511

[B257] Wang S. Jiang M. Duchesne X. M. Laugeson E. A. Kennedy D. P. Adolphs R. (2015). Atypical visual saliency in autism spectrum disorder quantified through model-based eye tracking. *Neuron* 88 604–616. 10.1016/j.neuron.2015.09.042 26593094 PMC4662072

[B258] Weaver M. S. Workman G. Cardo-Vila M. Arap W. Pasqualini R. Sage E. H. (2010). Processing of the matricellular protein hevin in mouse brain is dependent on ADAMTS4. *J. Biol. Chem.* 285 5868–5877. 10.1074/jbc.M109.070318 20018883 PMC2820812

[B259] Weaver M. Workman G. Schultz C. R. Lemke N. Rempel S. A. Sage E. H. (2011). Proteolysis of the matricellular protein hevin by matrix metalloproteinase-3 produces a SPARC-like fragment (SLF) associated with neovasculature in a murine glioma model. *J. Cell. Biochem.* 112 3093–3102. 10.1002/jcb.23235 21688302 PMC3188378

[B260] Wilson S. C. White K. I. Zhou Q. Pfuetzner R. A. Choi U. B. Sudhof T. C. (2019). Structures of neurexophilin-neurexin complexes reveal a regulatory mechanism of alternative splicing. *EMBO J.* 38:e101603. 10.15252/embj.2019101603 31566781 PMC6856630

[B261] Xie W. L. Zheng H. L. Li H. H. Lu J. J. Xue S. G. Luo Y. (2022). Deficiency of glycosylated alpha-dystroglycan in ventral hippocampus bridges the destabilization of gamma-aminobutyric acid type A receptors with the depressive-like behaviors of male mice. *Biol. Psychiatry* 91 593–603. 10.1016/j.biopsych.2021.10.022 35063187

[B262] Xu B. Ho Y. Fasolino M. Medina J. O’Brien W. T. Lamonica J. M. (2023). Allelic contribution of Nrxn1alpha to autism-relevant behavioral phenotypes in mice. *PLoS Genet.* 19:e1010659. 10.1371/journal.pgen.1010659 36848371 PMC9997995

[B263] Yamagata K. (2021). Astrocyte-induced synapse formation and ischemic stroke. *J. Neurosci. Res.* 99 1401–1413. 10.1002/jnr.24807 33604930

[B264] Yan Q. Sage E. H. (1999). SPARC, a matricellular glycoprotein with important biological functions. *J. Histochem. Cytochem.* 47 1495–1506. 10.1177/002215549904701201 10567433

[B265] Yan Q. Weyn-Vanhentenryck S. M. Wu J. Sloan S. A. Zhang Y. Chen K. (2015). Systematic discovery of regulated and conserved alternative exons in the mammalian brain reveals NMD modulating chromatin regulators. *Proc. Natl. Acad. Sci. U S A.* 112 3445–3450. 10.1073/pnas.1502849112 25737549 PMC4371929

[B266] Yao Z. van Velthoven C. T. J. Nguyen T. N. Goldy J. Sedeno-Cortes A. E. Baftizadeh F. (2021). A taxonomy of transcriptomic cell types across the isocortex and hippocampal formation. *Cell* 184 3222–3241 e3226. 10.1016/j.cell.2021.04.021 34004146 PMC8195859

[B267] Yue W. Yang Y. Zhang Y. Lu T. Hu X. Wang L. (2011). A case-control association study of NRXN1 polymorphisms with schizophrenia in Chinese Han population. *Behav. Brain Funct.* 7:7. 10.1186/1744-9081-7-7 21477380 PMC3080281

[B268] Yuzaki M. (2018). Two classes of secreted synaptic organizers in the central nervous system. *Annu. Rev. Physiol.* 80 243–262. 10.1146/annurev-physiol-021317-121322 29166241

[B269] Zaccaria M. L. Di Tommaso F. Brancaccio A. Paggi P. Petrucci T. C. (2001). Dystroglycan distribution in adult mouse brain: A light and electron microscopy study. *Neuroscience* 104 311–324. 10.1016/s0306-4522(01)00092-6 11377836

[B270] Zeng L. Zhang P. Shi L. Yamamoto V. Lu W. Wang K. (2013). Functional impacts of NRXN1 knockdown on neurodevelopment in stem cell models. *PLoS One* 8:e59685. 10.1371/journal.pone.0059685 23536886 PMC3607566

[B271] Zhang C. Y. Xiao X. Zhang Z. Hu Z. Li M. (2022). An alternative splicing hypothesis for neuropathology of schizophrenia: Evidence from studies on historical candidate genes and multi-omics data. *Mol. Psychiatry* 27 95–112. 10.1038/s41380-021-01037-w 33686213

[B272] Zhang C. Atasoy D. Arac D. Yang X. Fucillo M. V. Robison A. J. (2010). Neurexins physically and functionally interact with GABA(A) receptors. *Neuron* 66 403–416. 10.1016/j.neuron.2010.04.008 20471353 PMC3243752

[B273] Zhang P. Lu H. Peixoto R. T. Pines M. K. Ge Y. Oku S. (2018). Heparan sulfate organizes neuronal synapses through neurexin partnerships. *Cell* 174 1450–1464 e1423. 10.1016/j.cell.2018.07.002 30100184 PMC6173057

[B274] Zhang W. Rohlmann A. Sargsyan V. Aramuni G. Hammer R. E. Sudhof T. C. (2005). Extracellular domains of alpha-neurexins participate in regulating synaptic transmission by selectively affecting N- and P/Q-type Ca2+ channels. *J. Neurosci.* 25 4330–4342. 10.1523/JNEUROSCI.0497-05.2005 15858059 PMC6725120

[B275] Zheng M. Bao N. Wang Z. Song C. Jin Y. (2025). Alternative splicing in autism spectrum disorder: Recent insights from mechanisms to therapy. *Asian J. Psychiatr.* 108:104501. 10.1016/j.ajp.2025.104501 40273800

[B276] Zinebi F. Russell R. T. McKernan M. Shinnick-Gallagher P. (2001). Comparison of paired-pulse facilitation of AMPA and NMDA synaptic currents in the lateral amygdala. *Synapse* 42 115–127. 10.1002/syn.1107 11574948

[B277] Zweier C. de Jong E. K. Zweier M. Orrico A. Ousager L. B. Collins A. L. (2009). CNTNAP2 and NRXN1 are mutated in autosomal-recessive Pitt-Hopkins-like mental retardation and determine the level of a common synaptic protein in Drosophila. *Am. J. Hum. Genet.* 85 655–666. 10.1016/j.ajhg.2009.10.004 19896112 PMC2775834

